# Modelling the impact of climate on cholera: a case study of Kolkata

**DOI:** 10.1038/s41598-026-51415-z

**Published:** 2026-05-10

**Authors:** Debbie Shackleton, Shanta Dutta, Suman Kanungo, Alok Deb, Theo Economou

**Affiliations:** 1 Centre for Water Systems, University of Exeter, Exeter, United Kingdom; 2 ICMR - National Institute for Research in Bacterial Infections, Kolkata, India

**Keywords:** Computer modelling, Bacterial infection

## Abstract

Cholera is highly climate sensitive, however previous attempts to model its future under climate change have been limited to statistical analyses. Mechanistic models are an essential addition because they permit a deeper understanding of the complex feedback loops involved in infectious disease transmission, allowing for better modelling of potential scenarios such as interventions or changes in pathogen dynamics. We compare four mathematical models with differing assumptions of climate sensitivity and fit them to a cholera dataset from Kolkata, India using MCMC. We then use bias-corrected climate projections of temperature and rainfall from 10 independent global climate models to produce climate-based cholera projections for the period 2080–2099. Using both temperature and rainfall as inputs, the best performing model recreates seasonal patterns highly effectively. Future projections suggest an average increase in cholera cases ranging from 81% − 150% due to climate change by 2080–2099 with earlier peaks in the infection cycle likely due to heightened transmission rates earlier in the year. Sensitivity analysis reveals that uncertainties in parameters related to the contact rate and water dynamics have the greatest impact on model projections, suggesting that these factors are critical for refining future predictions. While our mechanistic model demonstrates the potential to project cholera dynamics under future climate scenarios, projections remain sensitive to key knowledge gaps including epidemiological parameters and effects of temperature on bacterial growth. Addressing these limitations through improved environmental observations and more detailed process representation will be essential for refining future climate-cholera projections and informing long-term control strategies.

**Supplementary Information:**

The online version contains supplementary material available at 10.1038/s41598-026-51415-z.

## Introduction

Cholera is highly climate-sensitive with a well-documented susceptibility to meteorological variables, particularly temperature and rainfall. Transmission is often associated with higher temperatures due to the preference of V. cholerae for warmer waters. The relationship with rainfall is more intricate and often highly region specific. High cholera transmission has been linked to both droughts^1, 2, 3^, which are thought to concentrate pathogenic bacteria in water sources, as well as floods, which increase.

human contact with contaminated water^2, 4, 5^. Given these sensitivities, climate change is expected to have a significant influence on the disease.

Numerous studies have examined the impact of climate change on specific infectious diseases, such as dengue^6, 7, 8^ and malaria^9, 10, 11^. However, comparatively few studies have explored this issue in relation to cholera. Early work by Nasr-Azadani et al^12^. linked global climate model projections to cholera outbreaks indirectly by using regression to model the influence of future climate-driven changes in precipitation on river discharge in the Bengal Delta. More recent studies have applied statistical and machine learning approaches more directly. For example Asadgol et al^13^. utilized artificial neural networks to evaluate the influence of climate change on cholera in Iran, while Kruger et al^14^. employed a random forest model to predict spatial changes in cholera risk in Bangladesh by 2050 using projected climate variables. Although these approaches can capture complex relationships involving numerous variables, they remain fundamentally empirical and do not explicitly represent the biological and environmental mechanisms driving cholera transmission. As a result, they cannot explain why projected changes may occur or identify the most effective interventions to disrupt these processes and prevent future transmission.

By incorporating processes of disease transmission, mechanistic models can facilitate a greater understanding of the complex feedback loops and non-linear processes typically present in infectious disease transmission^15^, as well as providing a vital tool with which to model potential scenarios such as interventions or changes to pathogen dynamics. In this article, we will propose four mathematical models describing cholera transmission and its relationship with climate, and compare their effectiveness at explaining cholera case data in our case study of Kolkata, India. We will then incorporate CMIP6 climate projections into the best performing model to produce cholera projections for the period of 2080–2099, under a specific climate scenario. Specifically, we will estimate what is the expected influence of a changing climate on cholera, conditionally on *all other things being equal*. In this way, we aim to develop an explicit and quantifiable understanding of cholera dynamics and the influence of climate through the first mechanistic cholera-climate model.

## Materials and methods

### Ethics approval and consent to participate

All ethical considerations and guidelines relating to the analysis of human epidemiological datasets were followed, namely full anonymization and aggregation of patient data beyond plausible identifiability. Informed consent was obtained from all subjects and/or their legal guardians. The study was approved by the Indian Council of Medical Research (ICMR). The research was conducted in accordance with the principles of the Helsinki Declaration.

### Epidemiological data

The cholera dataset consists of stool samples from diarrhoeal patients who reported to the Infectious Disease Hospital (IDH) in Kolkata, collected through their diarrheal surveillance system over a 21-year period (1999–2019), from the Indian Council of Medical Research - National Institute of Cholera and Enteric Diseases (ICMR-NICED). The surveillance involved testing every fifth patient on two randomly chosen days each week (about 6% of total patients) for various pathogens, including O1 and O139 Vibrio cholerae (*V. cholerae*). We extracted data on samples that tested positive for either O1 or O139 *V. cholerae*, specifically from patients residing in the Kolkata Municipal Corporation (KMC) area.

### Model definitions

**Fig. 1:**
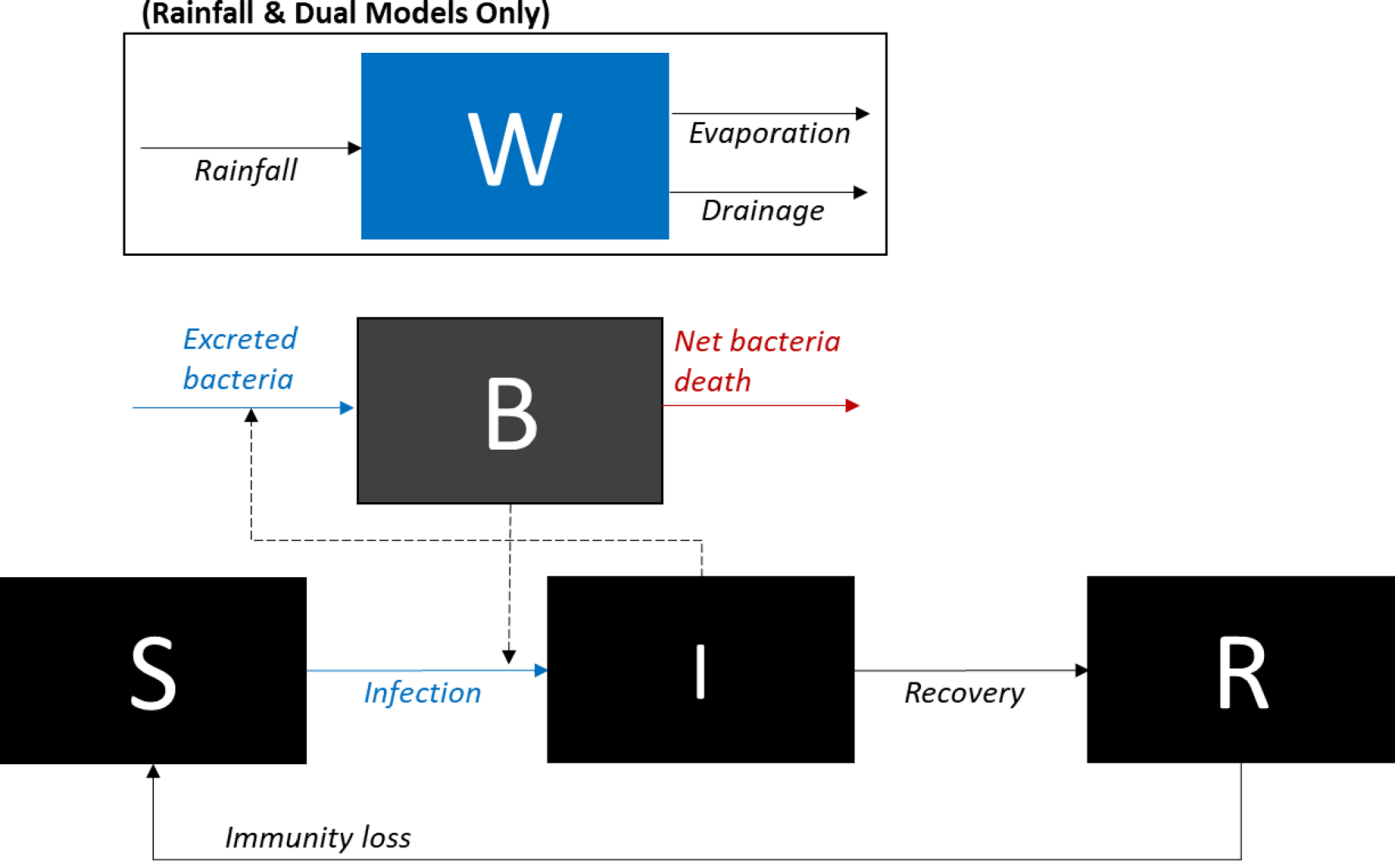
Diagram showing shared basic model structure. (S) tracks the proportion of the population which is susceptible, (I) infected, and (R) recovered. (B) tracks the population of pathogenic *V.cholerae* in the local aquatic environment, and (W), the volume of water in the local aquatic environment. Solid arrows denote transitions (inflow/outflow) of units from each compartment. Dashed arrows show where the state of one compartment influences a particular rate of transition. Transitions in red are temperature sensitive in the temperature and dual models. Transitions in blue are sensitive to water volume (and hence rainfall) in the rainfall and dual models. Birth and death are not shown for clarity.

In this study, we analysed four models of cholera transmission based on differing assumptions of climate forcing. The consideration of multiple models acts as a sensitivity analysis to test and justify the relevance of each included climate forcing variable. The four models (Fig. 1) are briefly described below, a more comprehensive description can be found in Supplementary Information S1. A summary of model parameters is provided in Table 1.

#### Base model

The base model was developed as a base case for comparison against the other three climate-driven models. It is not driven by any external forcing factor and so any emergent patterns in cases can be attributed to intrinsic dynamics. The human population is considered stable, hence the human birth and death rate $\:(\mu)$ are set as equal. The count of pathogenic *V. cholerae* in the urban aquatic environment (B) is modelled explicitly. These bacteria are assumed to be excreted into the environment by infectious humans with a constant rate $\:\mathop \in \limits^\prime$. Bacterial count tends toward environmental carrying capacity (net bacterial death) according to a logistic growth rate

$$- r\hat B\left( {1 - \hat B} \right)$$

where $\:\widehat{B}$ represents bacterial count relative to carrying capacity.

#### Temperature model

The temperature model introduces climate forcing through a daily temperature input. Temperature is assumed to influence cholera transmission indirectly via favourable conditions for *V. cholerae.* Specifically, bacterial dynamics are represented using a temperature-dependent logistic growth formulation in which both the intrinsic growth rate ($\:r$) and environmental carrying capacity $\:(C)$ are scaled by a temperature factor $\:{T}_{f}$ previously applied to other marine bacterial populations^16^.

$$\:Net\:bacteria\:death{=rT}_{f}\widehat{B}\left(1-\frac{\widehat{B}}{{T}_{f}}\right)$$

where $\:{T}_{f}={\left(\frac{Temp}{Tem{p}_{mean}}\right)}^{\alpha\:}$ $\:Tem{p}_{mean}$ represents the mean daily temperature across the observational period and $\:\alpha\:$ is a unitless parameter describing the sensitivity of bacterial dynamics to temperature. In this way, we make the implicit assumptions that warmer temperatures increase both the rate of bacterial growth^17, 18^ and sustained environmental abundance^17^ .

#### Rainfall model

The third model incorporates climate forcing through daily rainfall via an additional compartment, $\:W$, which represents the volume of surface water within the urban environment (e.g. ponds and canals). This approach follows earlier cholera modelling frameworks proposed by Pascual et al.^19^. The system is assumed to exist in one of two possible hydrological states: flooded or non-flooded. Flood conditions occur when water volume exceeds a threshold $\:{W}_{min}$, representing the maximum water storage capacity per unit area. For simplification, water volume is normalised in the model to $\:\widehat{W}=W/{W}_{min}$.

The dynamics of $\:\widehat{W}$ are described using a simplified water-balance model consisting of a single inflow (daily rainfall) and two outflow processes. A continuous outflow $\:(E\widehat{W})$represents combined evaporation and seepage losses. When $\:\widehat{W}>1$ (i.e. the flooded state), an additional, faster drainage term $\:D{\widehat{W}}_{flood}$ becomes active to represent removal of excess floodwater defined by $\:{\widehat{W}}_{flood}=\widehat{W}-1$ (see S1 for further details).

Variations in water volume are assumed to influence cholera transmission through two mechanisms. First, infection risk depends on bacterial concentration rather than total bacterial abundance^4^; consequently, the force of infection is assumed to scale with bacterial concentration ($\:\widehat{B}/\widehat{W}$). Second, flooding is assumed to increase transmission through disruption of sanitation systems and increased human contact with contaminated water^20^. This effect is represented by a flood amplification factor,

$$\:{F}_{f}=1+\widehat{\omega\:}{\widehat{W}}_{flood}$$

The resulting infection rate is therefore,

$$\:\widehat{\beta\:}{F}_{f}\frac{\widehat{B}}{\widehat{W}}$$

#### Dual model

The final model combines the assumptions of the Temperature and Rainfall models to provide a model sensitive to both parameters and can hence be described by the following set of coupled differential equations provided below.

$$\:\left\{ {\begin{array}{*{20}{c}} {\frac{{dS}}{{dt}} = \mu \:\left( {I + R} \right) - \beta {\:_2}\frac{B}{W}{F_f}S + \rho \:R}\\ {\:\frac{{dI}}{{dt}} = \beta {\:_2}\frac{B}{W}{F_f}S - I\left( {\mu \: + \eta \:} \right)\:\:\:\:\:\:\:\:\:\:\:\:\:}\\ {\:\frac{{dR}}{{dt}} = \eta \:I - R\left( {\mu \: + \rho \:} \right)\:\:\:\:\:\:\:\:\:\:\:\:\:\:\:\:\:\:\:\:\:\:\:\:}\\ {\:\frac{{dB}}{{dt}} = \in {F_f}I + r{T_f}B \cdot \:\left( {1 - \frac{B}{{{T_f}C}}} \right)\:\:\:\:}\\ {\:\frac{{dW}}{{dt}} = Rain\left( t \right) - D{W_{flood}} - EW} \end{array}} \right.$$

**Table 1:** Model parameters.

Model parameters	Description	Units	Prior distribution
$\:\boldsymbol{\mu\:}$	Birth/death rate	day^− 1^	4.15 × 10^− 5^ *
$\:\boldsymbol{\eta\:}$	Recovery rate	day^− 1^	0.2 *
$\:\boldsymbol{P}$	Proportion of infections reported to ID hospital	Dimensionless	$\:\:Beta\left(\mathrm{2,30}\right)$
$\:{\stackrel{\prime }{\boldsymbol{\beta\:}}}_{1}$	Relative contact rate between humans and bacteria	day^− 1^	$\:\:Beta\left(\mathrm{1,1}\right)$
$\:\stackrel{\prime }{{\boldsymbol{\beta\:}}_{2}}$	Relative contact rate between humans and infected water	day^− 1^	$\:\:Beta\left(\mathrm{1,1}\right)$
$\:\boldsymbol{\rho\:}$	Rate of immunity loss	year^− 1^	$\:\:Gamma\left(\mathrm{3,0.5}\right)$
$\mathop \in \limits^\prime$	Relative excretion into environment rate	day^− 1^	$\:\:Unif\left(\mathrm{0,1000}\right)$
r	Growth rate of Vibrio Cholerae	day^− 1^	$\:\:Beta\left(\mathrm{1,1}\right)$
$\:\boldsymbol{\alpha\:}$	Temperature sensitivity parameter	Dimensionless	$\:\:Unif\left(\mathrm{0,50}\right)$
$\:\stackrel{\prime }{\boldsymbol{\omega\:}}$	Flood sensitivity parameter	Dimensionless	$\:\:Unif\left(\mathrm{0,200}\right)$
$\:{\boldsymbol{W}}_{\boldsymbol{m}\boldsymbol{i}\boldsymbol{n}}$	Water volume at which ‘flooding’ occurs	$\:{m}^{3}/{m}^{2}$	$\:\:Gamma\left(\mathrm{3,0.005}\right)$
$\:\boldsymbol{D}$	Relative flood drainage rate	day^− 1^	$\:\:Beta\left(\mathrm{2,30}\right)$
$\:\boldsymbol{E}$	Evaporation rate	day^− 1^	$\:\:Beta\left(\mathrm{1,100}\right)$
Size	Dispersion parameter	Dimensionless	$\:\:Gamma\left(\mathrm{0.1,0.1}\right)$

See Supplementary Material S3 for derivation of prior distributions.

### Model parameterisations

Each model contained between four and nine unknown parameters that required inference from a relatively limited and autocorrelated dataset, introducing substantial uncertainty into model estimation. The nonlinear and partially observed structure of the transmission models further required a flexible estimation approach capable of inferring parameters indirectly from reported case data while efficiently exploring a high-dimensional parameter space. Here, we turn to the Bayesian learning approach Markov Chain Monte Carlo (MCMC)^21^ which involves assuming a statistical model, where the disease counts in the data are assumed to be driven by each of the four transmission models. A probability distribution is assumed to explain the discrepancy between the mechanistic models and the data (e.g., noise in the data, imperfect modelling assumptions etc.) (See Supplementary Material S3 for further details).

For each model, four independent MCMC chains were run to assess convergence to a common posterior distribution. Each chain was executed for a total of 20,000 iterations, with the first 18,000 iterations discarded as burn-in to remove dependence on initial parameter values. Convergence diagnostics were used to assess stable posterior sampling across chains following burn-in (Supplementary Material S4).

Prior probability distributions were specified for each parameter, where this can either be diffuse (uninformative) or informative, which is the way that Bayesian inference allows the user to include scientific knowledge in the estimation. A literature search was undertaken to inform prior specification where possible, while diffuse priors were adopted in cases where limited prior information was available. (Table 1; full justification provided in Supplementary Material S3). Compatibility between assumed priors and observed data was evaluated using prior predictive checks, in which model simulations generated from parameters sampled solely from the prior distributions were compared with observed epidemiological dynamics (Supplementary Material S4). These checks permitted validation that the priors produced realistic outbreak magnitudes and seasonal behaviour prior to model fitting.

As the true initial conditions of the system were unknown, arbitrary values were assumed and simulations were preceded by a 10-year warm-up period to allow convergence towards endemic equilibrium. Sensitivity analyses of the initial conditions was performed to verify that model outputs were largely insensitive to assumptions regarding initial conditions following this warm-up period (Supplementary Material S4).

### Model comparison metrics

In this analysis, we measured model performance by using two metrics. The first is the Watanabe-Akaike Information Criterion (WAIC), a metric for comparing competing models. This metric estimates the out-of-sample predictive capacity of each model without the need for partitioning data into testing and training. Overfitting is allowed for via the inclusion of a penalty for model complexity. A lower WAIC score indicates better predictive capacity.

Second, the ability of each model to predict interannual differences in the magnitude of seasonal outbreaks is compared using Spearman’s Rank correlation. For each model the correlation coefficient is measured between the simulated and observed values of maximum cholera cases occurring during a single month each year, for each summer (March-June) and monsoon (July-December) cholera seasons. Statistical significance is defined as *p* < 0.05.

## Climate projections

### Projected climate inputs

Projections of future climate variables are produced by General Circulation Models (GCMs), numerical models simulating global coupled physical processes in the atmosphere, ocean, cryosphere and land surface. We accounted for uncertainty in climate projections by using a selection of 10 independent GCMs in our model projections. In addition, we also consider projections under three Shared Socioeconomic Pathways (SSPs): a best-case scenario (SSP1-2.6), a middle-of-the-road scenario (SSP2-4.5), and a worst-case scenario (SSP5-8.5). Under current global policy trajectories, SSP2-4.5 is generally considered the most plausible pathway^22, 23^, while SSP1-2.6 and SSP5-8.5 are included here to represent lower and upper bounds of radiative forcing in order to assess the sensitivity of cholera projections to alternative climate futures.

It is largely accepted that GCMs are subject to persistent, systematic biases^24, 25^. Further, due to limits in computing power, GCMs generally produce outputs at coarse spatial resolutions, ranging from 0.94° to 2.5° within the selected models (Table S2.1). This is a particular issue in the tropics where precipitation patterns can vary significantly over short distances^26^ and the variation in spatial resolution compromises meaningful comparison between outputs. To overcome these issues, we applied the Change Factor Methodology (CFM), a simple downscaling and bias-correction methodology commonly used in impact studies^27^, to each GCM and SSP (see supplementary information S2). While more sophisticated bias-correction techniques exist (e.g., trend-preserving quantile mapping), CFM was considered appropriate here because the rainfall forcing in the transmission model is represented using a rolling mean of daily rainfall, reducing sensitivity to extreme precipitation values that such methods are designed to correct.

### Cholera projections

Projections for proportional increases in cholera infections were produced by running the most effective cholera model using the bias-corrected climate projections for 2070–2099 as inputs. As in the calibration process, projection simulations were run for 30 years, with the first 10 years (2070–2079) discarded to allow the model to reach endemic equilibrium.

Uncertainty was incorporated into the model projections via two avenues. First, through uncertainty in parameter values as characterized by their posterior distributions shown in Figure S4.10. Second, through uncertainty in climate forcing which is characterized by the variation in outputs of the selected GCM models (Table S2.1), resulting in a total of 11 uncertain inputs.

Input uncertainty was propagated to output uncertainty in cholera projections using a Monte Carlo framework consisting of 10,000 simulations. Due to the large (11-dimension) sample space, model parameters for each simulation were sampled using Latin Hypercube Sampling (LHS) in order to fully represent input uncertainty within computational constraints. The model output and its uncertainty were then described by the median of all 10,000 simulations and the 95% confidence interval.

### Sensitivity analysis of climate projections

A sensitivity analysis was conducted to attribute uncertainty in the model outputs to uncertainty in the model inputs. We identified the contribution of each input parameter the overall model response using the Sobol’ method^28^, a variance-based global sensitivity analysis. Two Sobol’ indices are used in this analysis: first-order indices which describe the impact on model output of varying each parameter alone, ignoring interactions with other variables; and total-order indices, which accounts for the total variance in the output caused by input variation accounting for both individual effects and interaction effects.

As with the uncertainty analysis, 11 uncertain parameters were included in the sensitivity analysis: the 10 uncertain model parameters whose probability distributions are described by their posterior distributions given in Figure S4.10. Uncertainty in climate inputs was again represented by variation in GCM outputs. For simplification and increased interpretability of results, ‘model output’ in the context of the sensitivity analysis was defined as a scalar value denoting the total number of cholera infections incurred during the simulation period.

## Results

### Model selection

**Fig. 2:**
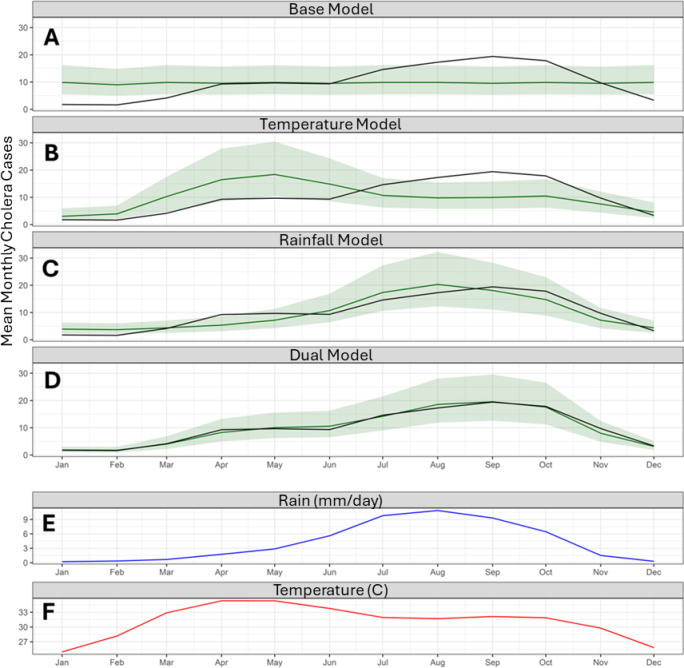
Mean monthly cholera case during reference period for Base (**A**), Temperature (**B**), Rainfall (**C**) and Dual (**D**) models. Median posterior values are indicated in green with shading representing 95% credible interval. Case values are shown in black. For context, mean monthly rainfall (**E**) and Temperature (**F**) are shown below.

**Fig. 3:**
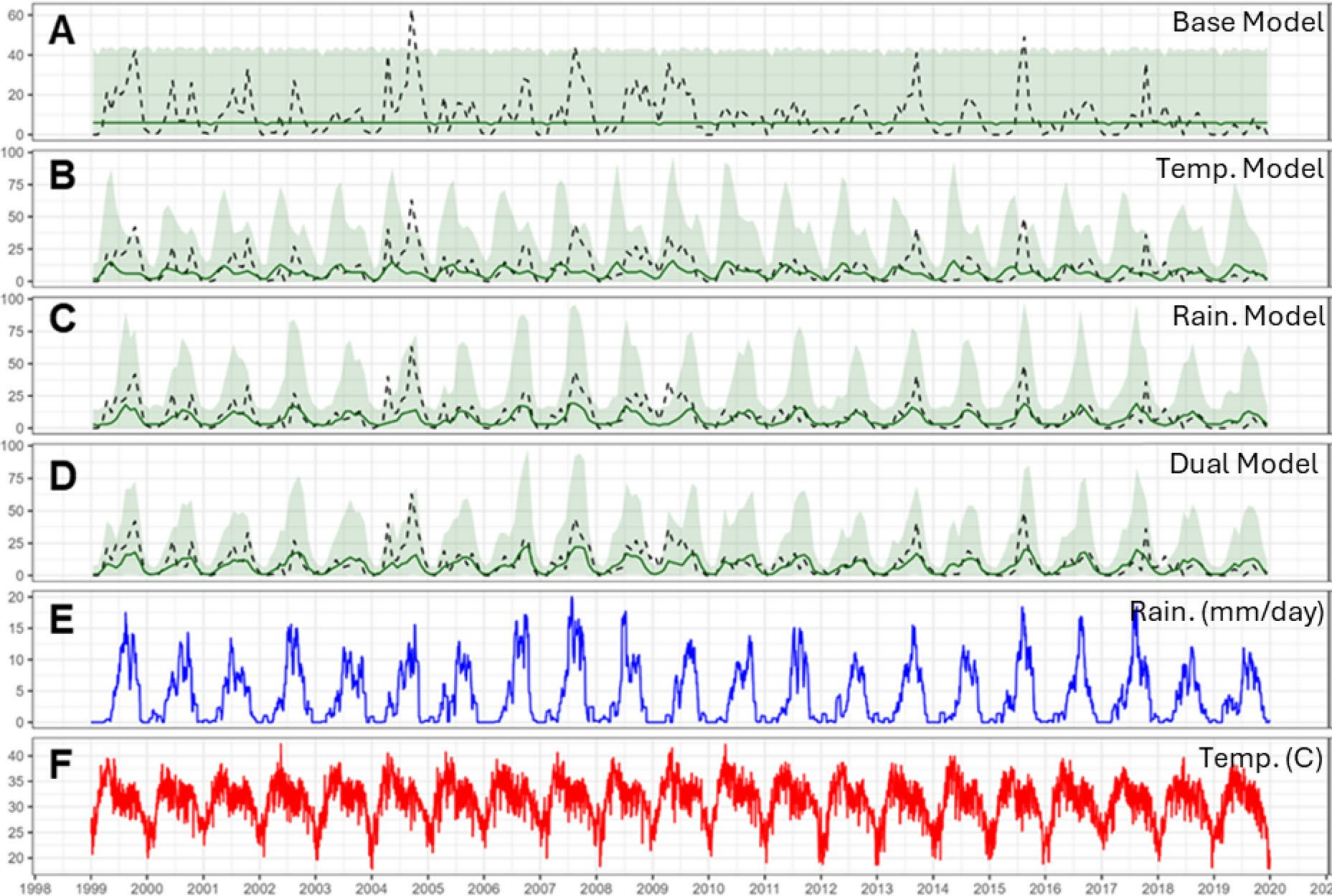
Full time series simulation of monthly cholera cases according to Base (**A**), Temperature (**B**), Rainfall (**C**) and Dual (**D**) models. Dotted black line represents recorded data, green line median posterior simulated values and shaded area denotes 95% credible interval. 28-day smoothed rainfall (**E**) and daily temperature (**F**) are given below for reference. Note that the y-axis scale differs among panels **A**-**D**.

**Fig. 4:**
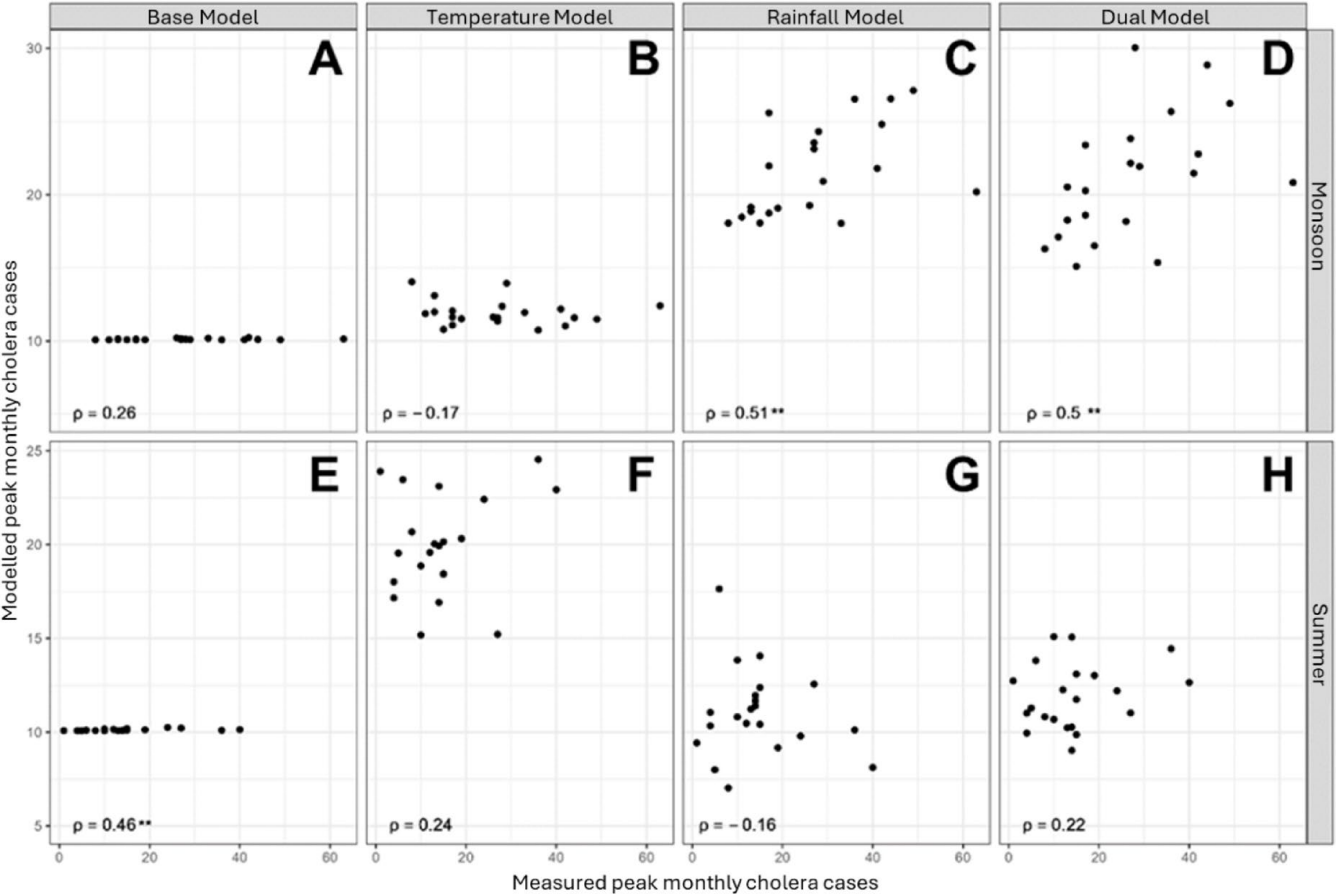
Relationship between measured and modelled peak monthly cholera cases for each transmission model. Panels **A**–**D** show correlations during the monsoon season, while panels **E**–**H** show correlations during the summer season, for the Base, Temperature, Rainfall, and Dual models, respectively. Each point represents the peak monthly cholera incidence for an individual year. Spearman’s rank correlation coefficient (ρ) is shown in the lower-right corner of each panel, with statistically significant correlations (*p* < 0.05) indicated by **. Higher correlation values indicate improved model skill in reproducing interannual variability in seasonal outbreak magnitude. Note the Y-axis differs between panels **A**-**D** and **E**-**H**.

Simulated cholera cases for each of the four models over the reference period are shown in Figs. 2 and 3 with shaded areas representing uncertainty propagated from the posterior distributions of the estimated model parameters. The base model showed no significant seasonal variation and failed to capture the peaks and troughs in the data (Figs. 2A and 3A), suggesting that intrinsic dynamics alone are insufficient to explain seasonal variation. This poor predictive performance is evidenced by the lower WAIC score of all models tested (Table 2) and inability to predict the magnitude of either summer or monsoon peak (Fig. 4A, E).

**Table 2:** Model performance comparison.

Model	WAIC score	Ranking
Dual	1549.8	1
Rainfall	1599.2	2
Temperature	1629.0	3
Basic	1675.6	4

By incorporating temperature sensitivity into the ODE system, the Temperature Model successfully predicted the observed increase in cholera cases from March to May during the summer season, responding to rising temperatures (Fig. 2B, F). The model demonstrated some accuracy in predicting the relative magnitude of the summer peak (though not the monsoon peak) (Fig. 4B, F); however, the interannual variations in peak sizes were significantly less diverse than those observed in the actual data (Fig. 3B), potentially due to low interannual variation of temperature (Fig. 3F). The model failed to predict the rise in cases from June to September and significantly overestimated the summer peak, likely as a compensatory measure for its inability to predict monsoon-related cases. Interestingly, the Temperature Model predicted a small second peak in October, likely driven by a slight temperature increase during this period.

Conversely, the Rainfall Model successfully predicted the monsoon peak, although it was around a month early in its prediction (Fig. 2C). While the model did not predict a summer peak in its seasonal profile, nor in interannual variation of magnitude (Fig. 4G) it successfully anticipated a rise in cases around March, prior to the onset of monsoon rains (Fig. 2E). This can potentially be attributed to the dependence on bacterial concentration (rather than count), which causes the force of infection to increase in response to decreased water volume. Despite its inability to predict a summer peak, the Rainfall Model outperformed the Temperature Model in overall predictive capacity, with a WAIC value of 1599.2 compared to the Temperature Model’s 1629.0, and showed a much improved seasonality profile. Furthermore, the model demonstrated a reasonable capacity to predict the magnitude of the monsoon peak (Fig. 4C), with a significant rank correlation of ρ = 0.51 between modelled and measured values.

Among all models, the Dual Model demonstrated the highest predictive capacity (Table 2), uniquely predicting a dual-peak seasonality in most years (Fig. 3D). Similar to the Rainfall Model, the Dual Model showed a reasonable ability to predict the magnitude of the monsoon peak. However, while a small positive correlation is evident regarding the relative magnitude between simulated and observed summer peaks, the effect is not significant (Fig. 4H).

None of the models were able to reasonably predict the magnitude of interannual variation in cholera peaks (Fig. 3A-D) despite defined interannual variation in both temperature and rainfall (Fig. 3E, F). Despite this, the predictions of the Dual Model align remarkably well with the data when considering monthly averages (Fig. 2D), indicating its high proficiency in capturing seasonal cholera patterns in Kolkata, including the timing and relative size of each seasonal epidemic. Further, the Dual Model predicts the timing of the monsoon peak more accurately than the Rainfall Model (Fig. 2), suggesting that the post-monsoon temperature increase could, in addition to increased rainfall, partly explain the October peak. Due to its success in predicting cholera cases, we retained the Dual Model for use in developing future projections.

### Future projections

**Fig. 5:**
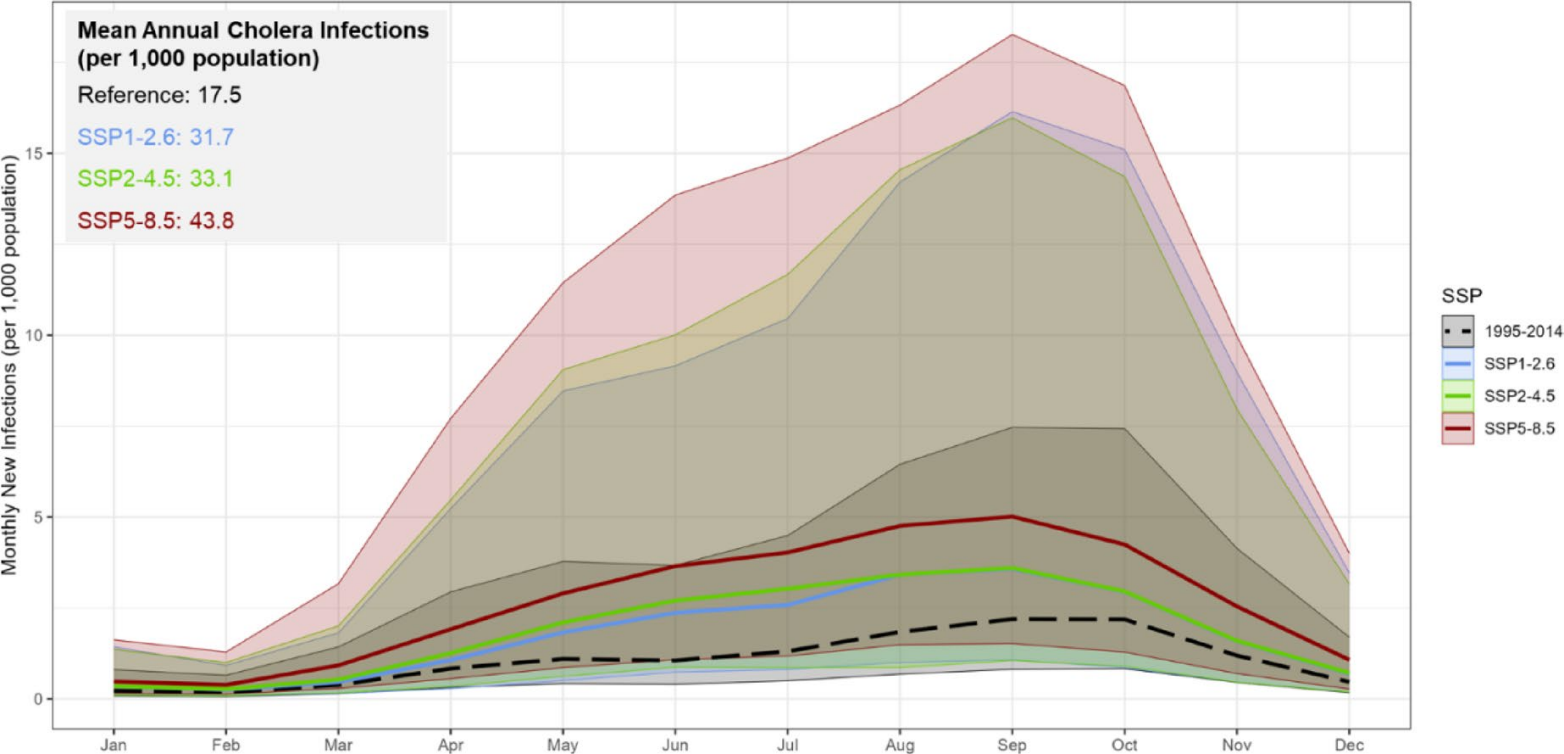
Mean monthly projected cholera infections per 1,000 population for the future period 2080–2099 are represented by the solid lines. Blue represents the SSP1-2.6 scenario, green represents SSP2-4.5, red represents SSP5-8.5. Dashed black line shows the mean simulated cholera monthly infections for the reference period 1995–2014. Shaded bands provide 95% credible intervals in respective colours.

The seasonal projections of mean monthly cholera infections are shown in Fig. 5. Mean monthly projected cholera infections per 1,000 population for the future period 2080–2099 are represented by the solid lines. Blue represents the SSP1-2.6 scenario, green represents SSP2-4.5, red represents SSP5-8.5. Dashed black line shows the mean simulated cholera monthly infections for the reference period 1995–2014. Shaded bands provide 95% credible intervals in respective colours., where the credible intervals represent uncertainty from both posterior parameter estimates and variability in the climate projections. A clear increase in projected cholera incidence is evident across all three SSP scenarios during the future period (2080–2099) compared with the reference period (1995–2014). The model simulation suggests an average increase in cholera infections of 81% under the SSP1-2.5 scenario, 89% under SSP2-4.5, and 150% under SSP5-8.5.

**Fig. 6:**
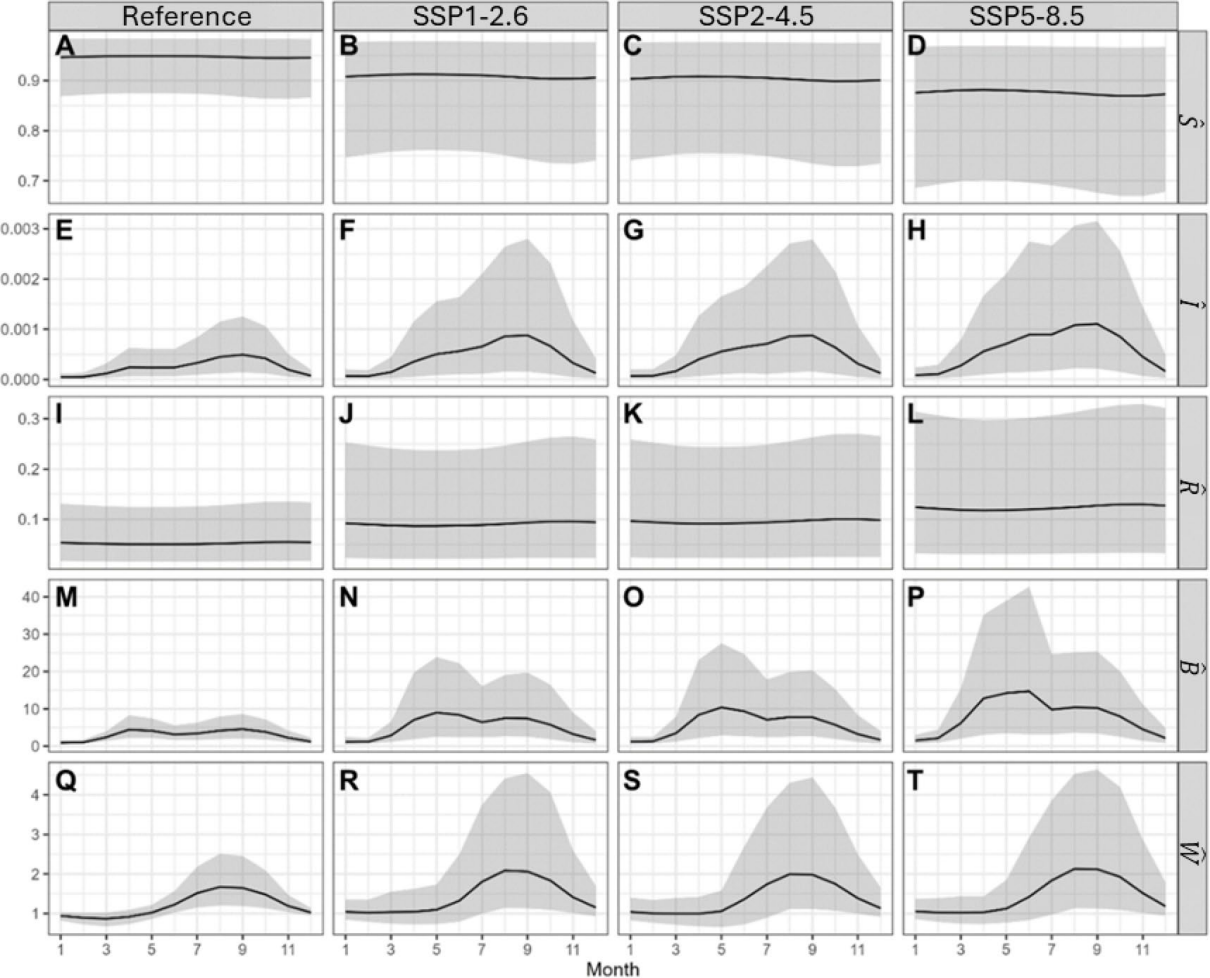
Monthly mean simulated dynamics of normalized model state variables for the historical reference period (1999–2019) and future climate scenarios (2080–2099). Columns correspond to the reference simulation and SSP1-2.6, SSP2-4.5, and SSP5-8.5 scenarios. Rows show model variables: susceptible population ($\:\widehat{\boldsymbol{S}}$; **A**–**D**), infected population ($\:\widehat{\boldsymbol{I}}$; **E**–**H**), recovered population ($\:\widehat{\boldsymbol{R}}$; **I**–**L**), environmental bacterial abundance ($\:\widehat{\boldsymbol{B}}$; **M**–**P**), and normalized water volume ($\:\widehat{\boldsymbol{W}}$; **Q**–**T**). Solid lines denote the mean across simulations, while shaded regions represent the 95% credible interval. All variables are dimensionless.

Table S4.2 shows the relative increase in expected mean monthly cholera cases compared with the reference period under each simulated SSP scenario. While expected mean cholera infections are projected to increase in all months of the year, the proportional increase is greatest in June for all scenarios, and lowest in Feb-March. This heterogeneity in projected changes to monthly cholera infections can also be witnessed in the change in seasonal patterns (Fig. 5). In the simulated results for the reference period there is a slight dual peak with a reduction in cases between May and June, a phenomenon also witnessed in the observed cholera case data for the reference period (Fig. 2). However, in each of the SSP scenarios the summer and monsoon peaks are not differentiated and instead form a continual increase in month-on-month cholera infections from February to March, though the rate of increase is slightly lessened between June-July. This may be due to the number of bacteria in the environment remaining high in May and June (Fig. 6M-P) owing to higher temperatures, mediating continued cholera transmission in the transition period between summer and monsoon peaks.

An interesting finding is that, despite the climate projections of monsoon rains occurring later in the year (Figure S2.4), future simulations of our transmission model project an earlier peak in cholera infections, from October to September in all three future simulations. This is likely due to a general increase in transmission leading to greater depletion in the susceptible population (Fig. 6A-D), and an increased immune population (Fig. 6I-L). This reduction in susceptible population may cause the effective reproduction number to fall below one (and therefore peak in cases (Fig. 6E-H)) earlier in the year causing an earlier epidemic peak.

The climate variables temperature and rainfall influence the force of cholera infection via changes in two modelled compartments: relative bacteria count, $\:\stackrel{\prime }{B}$, and relative urban surface water volume $\:\stackrel{\prime }{W}$. From Fig. 6M-T it is clear that the influence of climate has a notable effect on both of these variables, particularly $\:\stackrel{\prime }{B}$. With regards to $\:\stackrel{\prime }{W}$, all three SSP scenarios project greater flooding during the monsoon, however the difference in overall monsoon flooding projected under each SSP scenario is minimal (Fig. 6Q-T). Given that rainfall is only permitted to influence cholera transmission via the $\:\stackrel{\prime }{W}$ compartment, this suggests that the average 20% increase in cholera infections in the monsoon in the SSP5-8.5 scenario compared with the SSP1-2.5 and SSP2-4.5 scenarios is mediated by increased temperature rather than by increased rainfall.

### Model sensitivity

**Fig. 7:**
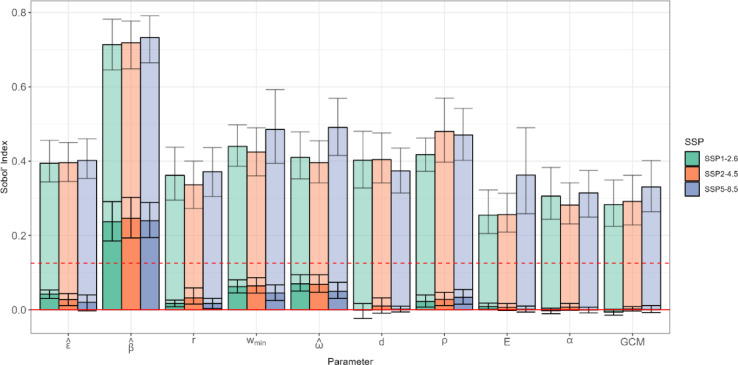
Sobol’ Indices of model inputs under three climate scenarios. Green represents SSP1-2.6 scenario, orange SSP2-4.5, and blue SSP5-8.5. First order effects (S_i_) are represented by solid colour, total order effects (T_i_) are shown as faded. Error bars represent 95% confidence interval and are given in full black for first over effects, and faded black for total order effects. Horizontal red lines represent indices of dummy parameters for first order effects (solid) and total order effects (dashed).

The Sobol’ indices for the first and total order effects of each input are presented in Fig. 7. The 95% confidence intervals for the first-order effects of $\:\mathop \in \limits^\prime$, $\:\stackrel{\prime }{\beta\:}$,$\:r$,$\:{W}_{min}$,$\:\stackrel{\prime }{\omega\:}$, and $\:\rho\:$ all lie above the dummy parameter (which has no influence on the model output), indicating that uncertainty in these parameters contributes significantly to the overall uncertainty in model projections. Among these, the contact rate parameter, $\:\stackrel{\prime }{\beta\:}$, exerts the greatest influence, accounting for approximately 24% of the variance in projected total cholera infections. Consequently, reducing uncertainty around the ‘true’ value of $\:\stackrel{\prime }{\beta\:}$ would most effectively improve the overall certainty in model predictions. From a practical perspective, the highlights the importance of better quantifying human-.

The total order indices are significant for all model parameters, indicating that all parameters are influential in the model output. Therefore, the model cannot be simplified by removing parameters without significantly altering the output. Notably, the total order effects are much greater than the first order effects for all variables, highlighting a strong influence of parameter interactions.

The sensitivities of each input parameter are largely consistent across climate scenarios. The most significant differences are seen in the increased sensitivities to certain water-related parameters; namely flood capacity $\:{W}_{min}$, relative flood factor $\:\stackrel{\prime }{\omega\:}$, and evaporation rate $\:E$. This could suggest that rainfall becomes more influential under the SSP5-8.5 scenario and highlights the potential importance of urban water management as a potential leverage point for reducing future cholera risk under climate change. However, given that none of these differences are statistically significant, no definitive conclusions can be drawn. The output uncertainty contributed by variance in climate projections increases with scenario radiative forcing. This is expected as variation between model projections increases with scenario radiative forcing (Figures S2.3, S2.4).

## Discussion

Our study demonstrates that while temperature is most important for explaining the summer peak and rainfall for the monsoon peak observed in Kolkata, both variables are essential for accurately modelling either peak. By testing multiple environmental models, we developed a model that effectively captures the seasonal profile of cholera using climate variables. Our findings indicate that climate variables can serve as strong predictors of the seasonal timing of cholera outbreaks. Additionally, our model shows early promise in predicting the seasonal magnitude of the cholera burden according to variation in climate, particularly the monsoon peak. However, interannual variation in cases was consistently underestimated in all models, suggesting outbreak severity may be influenced by factors beyond the modelled climate inputs, such as contamination of the local drinking water supply^29^.

Utilizing this novel model, we were able to produce projections for three 2080–2099 climate scenarios. Our results tentatively suggest a substantial increase in cholera infections with mean annual increases ranging from 81% under the most ambitious climate scenario and 150% under the most pessimistic. These findings align with the two other previous studies which have attempted to project the influence of climate on cholera outbreaks^13, 14^. Both concluded that increased temperatures and changes to rainfall patterns would likely lead to an increase in cholera transmission.

Previous studies examining cholera under climate change have primarily relied on either indirect environmental proxies or statistical relationships between climate variables and disease outcomes^12, 13, 14^. Although these approaches demonstrate that cholera risk is likely to increase under future climate scenarios, they do not explicitly represent transmission dynamics or the environmental feedbacks through which climate influences infection. In contrast, the present study integrates bias-corrected climate projections within a mechanistic transmission framework that explicitly links temperature and rainfall to bacterial dynamics, environmental water conditions, and host susceptibility. This process-based representation enables projected changes in cholera burden to be interpreted mechanistically, such as shifts in epidemic timing arising from climate-driven increases in transmission rather than inferred solely from statistical correlation. Our results broadly support earlier findings of increasing cholera risk under warming scenarios, but extend existing work by providing climate-change projections derived from an explicitly dynamical disease model, thereby enabling attribution of projected changes to underlying transmission processes and offering a framework suitable for future evaluation of intervention or adaptation scenarios.

These findings have important policy implications, particularly in the context of our Kolkata case study. Firstly, they add to a growing body of evidence that climate change is likely to elevate cholera risk in this region, primarily due to warmer waters that create more favourable conditions for V. cholerae. Secondly, our results indicate a potential shift from dual-seasonal outbreaks to a more continuous transmission period, with only a brief reduction in infections between December and February. This could reduce the window currently available for health systems to recover during the mid-year lull between peaks. A longer and more intense transmission season is also likely to increase cumulative infections and therefore population immunity, potentially reducing the cost-effectiveness of a vaccination strategy. Together, these insights underscore the critical need for sustained long-term investments in cholera control, particularly through improvements to Water, Sanitation and Hygiene (WASH) infrastructure to limit contamination of surface water bodies, flood control measures to reduce the spread of contaminated water, and strengthening the resilience of healthcare institutions^30^.

### Model limitations and future work

It has been said that a mathematical model is “no more, and no less, than clearly thinking about the problem at hand”^31^. In this context, we believe that one of the important contributions of this study lies in the identification of uncertainties and knowledge gaps in our current understanding of the climate-cholera relationship. Enhancements to the mechanistic understanding of this relationship can be pursued through two avenues: reduction in parameter uncertainty and improvements to constraints within the model structure.

The sensitivity analysis revealed that reducing uncertainty in the contact rate between humans and bacteria (β̂) would significantly improve the certainty of model projections. Although directly measuring this parameter is challenging, its uncertainty could be potentially reduced through intermediate calibration approaches. For instance, time-series data on *V. cholerae* concentrations in urban water sources would help constrain environmental transmission dynamics and reduce uncertainty in the human-environment contact process. From a practical perspective, this highlights the importance of strengthening environmental surveillance systems and integrating microbiological monitoring with epidemiological data collection in cholera-endemic cities. Significant output uncertainties also arise from more directly measurable parameters, such as the rate of immunity loss (ρ). Duration of protection conferred by natural cholera infection remains poorly understood^32^ and improved longitudinal studies of post-infection immunity would help refine prior estimates, decreasing overall uncertainty.

A potential structural simplification that may explain the model’s limited predictive skill for the summer peak lies in the parameterisation of the relationship between temperature and *V. cholerae* growth. Most aquatic bacteria exhibit a typical thermal performance curve, characterised by a unimodal response in which increasing temperature enhances growth rates up to an optimal threshold, beyond which further warming leads to declining growth and reduced population abundance^33^. Early laboratory studies from the 1980 s found increased growth of *V. cholerae* at temperatures up to 30 °C^17, 18^, a finding supported by more recent observations linking warmer temperatures to elevated case incidence^34^. Additionally, *Vibrio vulnificus*, which shares the same genus as *Vibrio cholerae*, has demonstrated higher growth rates at temperatures up to at least 37 °C^35^. However, evidence in current literature describing behaviour of *V. cholerae* at higher temperatures is scarce, and a comprehensive mathematical relationship between bacterial growth and temperature therefore remains elusive. Our climate analysis indicates average summer temperatures in Kolkata could reach up to 40 °C, well outside currently experimented conditions. The parameterisation adopted here assumes a monotonic increase in bacteria with temperature. and may therefore overestimate summer cholera burden, particularly under the warmest climate scenarios. Improved empirical understanding of high-temperature responses in *V. cholerae* would substantially enhance future mechanistic cholera–climate models.

Additionally, the models developed in this study utilize a highly simplistic water balance sub-model to account for the rainfall-cholera relationship which overlooks key factors such as topography, land use, drainage systems, and the effect of temperature on evaporation. Integrating a basic hydraulic sub-model could yield more accurate and mechanistic estimates of the relationship between rainfall and urban water dynamics, and consequently, cholera transmission, an approach successfully implemented for other climate-sensitive diseases such as malaria^36, 37^. This enhancement could also offer improved future projections by accounting for the effect of changes in land use or drainage infrastructure.

This study considers only the direct effects of changes to precipitation and temperature, omitting indirect pathways through which climate may modify cholera risk. Using our case study as an example, Kolkata’s low elevation and subsidence rate of over 13 mm/year make the city particularly vulnerable to sea-level rise^38^, which may exacerbate flooding through increased river overtopping and impaired drainage, while saltwater intrusion may further enhance *V. cholerae* persistence in aquatic environments^4^. A coupled hydraulic sub-model could be extended to represent these processes. Further, climate change is widely expected to result in a large number of ‘climate refugees’ from many South Asian countries, particularly from nearby Bangladesh, as well as internally displaced persons from within India^39, 40^. This could lead to significant migration to urban centres like Kolkata, increasing population density and straining water and sanitation infrastructure and thereby intensifying cholera risk^4^. Future modelling efforts could address these dynamics through explicit representation of population change using demographic projections aligned with SSP scenarios (e.g^41^.), enabling a more holistic assessment of climate-driven cholera risk.

An additional caveat to this study is that the epidemiological dataset used for model calibration records confirmed cholera cases rather than total infections. As a result, the data are subject to systematic case ascertainment bias, as the probability that an infected individual seeks hospital care may vary over time. For example, individuals may be more likely to seek treatment when the perceived threat of cholera is greater, such as following public health awareness campaigns, during the traditionally recognised “cholera season” associated with the monsoon, or during periods of economic hardship when malnutrition increases the risks associated with diarrhoeal illness. These behavioural and socioeconomic factors could influence the relationship between observed cases and underlying transmission, potentially introducing temporal biases in the reported case counts used for model fitting.

## Conclusion

Our study highlights the critical role of climate variables temperature and rainfall in shaping the seasonal dynamics of cholera in Kolkata. By developing an early mechanistic model of the effects of climate change on cholera transmission, we demonstrate that these environmental factors are not only strong predictors of the seasonal timing of outbreaks but also show potential in projecting the magnitude of cholera burdens, particularly during the monsoon season. Projections for future climate scenarios suggest a significant increase in cholera infections by the end of the century, underscoring the urgent need for long-term investments in cholera control programs.

Importantly, the modelling framework developed here offers key advantages for assessing climate-sensitive disease under future environmental change. Its mechanistic structure improves interpretability by linking projected changes in cholera burden to identifiable transmission processes, while explicitly representing both temperature- and rainfall-driven pathways that govern the regions dual seasonal outbreaks. Integration of CMIP6 climate projections enables consistent exploration of future climate uncertainty, and accompanying sensitivity analysis identifies the epidemiological and hydrological processes that most strongly influence projections. Together, these features provide a transparent and extensible framework for evaluating how climate change may alter cholera dynamics and for supporting future intervention and adaptation planning.

However, our findings also reveal several areas of uncertainty and structural simplifications within our current understanding of the cholera-climate relationship that need to be addressed. Key gaps include the relationship between temperature and *V. cholerae* growth, the effects of extreme temperatures, and the simplifications in modelling the rainfall-cholera relationship. Addressing these gaps through further research and model refinement will be crucial for improving the accuracy of cholera projections under changing climate conditions.

In light of these challenges, future efforts should focus on reducing parameter uncertainties, integrating more comprehensive sub-models, and considering indirect effects of climate change, such as rising sea levels and population displacement. A comprehensive understanding of the interconnected nature of public health with social, demographic, and environmental drivers of infectious diseases is critical. Enhanced collaboration between public health practitioners, climate scientists, civil engineers, social scientists, network modelers, and decision-makers is warranted. By addressing these additional factors and uncertainties, future research can continue to improve our understanding and response to the impacts of climate change on cholera transmission.

## Supplementary Information

Supplementary Information 1.

Supplementary Information 2.

Supplementary Information 3.

Supplementary Information 4.

## Data Availability

All climate data, both historical observations and future projections are publicly available. Details of how to access all sources are provided in Supplementary Materials S2. Epidemiological data is the property of the Indian Council of Medical Research (ICMR) and is not publicly available due to political, privacy and ethical concerns.
