# Supplementary material for: Modelling the impact of climate on cholera: a case study of Kolkata
Source: Sci Rep. 2026 May 10;16:21346. doi: 10.1038/s41598-026-51415-z (PMC13346884; doi:10.1038/s41598-026-51415-z)
Supplement: Supplementary file 1 — Supplementary Information 1. [file 41598_2026_51415_MOESM1_ESM.docx]

## S1 - Model Description

**Basic Model**

The ‘basic’ model was developed as a base case for comparison against the other three climate-driven models. It is not driven by any external forcing factor and so any emergent patterns in cases can be attributed to intrinsic dynamics. It also serves here to explain the common terms in the three climate-driven models as they are all extensions of the basic model. This model is defined by the set of coupled differential equations given in. The constituent elements are described in further detail below.

| $\left\{ \begin{aligned} \frac{dS}{dt}=\mu\left( I+R \right)-\beta_{1}BS+\rho R \\ \frac{dI}{dt}=\beta_{1}BS-I\left( \mu+\eta\right) \\ \frac{dR}{dt}=\eta I-R\left( \mu+\rho\right) \\ \frac{dB}{dt}=\epsilon I+rB\cdot\left( 1-\frac{B}{C} \right) \end{aligned} \right.$ |
| --- |

Where $\mu$ represents the birth and death rate of the population, individuals are born susceptible, and deaths can occur in S, I, and R compartments. Given the relative stability of the population, birth and death rates are assumed equal.

$$\boldsymbol{Infection rate=}\beta_{1}B\left( t \right)S(t)$$

The infection rate describes the rate at which susceptible individuals become infected. This is given as the product of the bacteria count in the environment $B(t)$, the availability of susceptibles $S(t)$, and the effective contact rate between susceptibles and bacteria, $\beta_{1}$, where ‘effective contact’ describes contact which results in infection. Units of $\beta_{1}$ are $bacteria^{-1}day^{-1}$

$$\boldsymbol{Recovery rate}=\eta I(t)$$

The recovery rate describes the rate of transition of individuals from the ‘infected’ to ‘recovered’ compartments. It is given as the product of the recovery rate, $\eta$, and the number of infected individuals $I(t)$. Units of $\eta$ are $day^{-1}$ and its reciprocal, $1/\eta,$is the duration of infection in days.

$$\boldsymbol{Rate of immunity loss}=\rho R(t)$$

Immunity is assumed temporary, with duration $1/\rho$ days. Once a recovered individual loses immunity, they return to the susceptible compartment. Units of $\rho$ are $day^{-1}$.

The bacteria count in the environment is influenced in two ways, via faecal shedding (excretion) by infected individuals and by natural bacterial processes.

$$\boldsymbol{Rate of bacteria excretion}=\epsilon I(t)$$

Where $\epsilon$ describes the average number of bacteria shed into the environment by a single person per day and has units $persons^{-1}day^{-1}$.

$$\boldsymbol{Natural Bacterial Processes}= rB\left( t \right)\cdot\left( 1-\frac{B(t)}{C} \right)$$

The ‘natural’ dynamics of pathogenic *V. cholerae* are described by a logistic differential equation, commonly used to describe bacterial populations [1], and has been previously utilised in a theoretical model of waterborne infections [2]. The intrinsic rate of change of bacterial population is defined by growth rate, $r$ (*unitless*) and carrying capacity $C$ (*bacteria*). In a bacteria-free environment, bacteria will increase exponentially, and slow as $B\left( t \right)$ approaches $C$. When bacterial count exceeds $C$ (due to excreted bacteria), the term becomes negative and tends towards $C$.

Positivity and Boundedness of Solutions

**Lemma 1.** *Given nonnegative initial conditions, the solutions (S(t), I(t), R(t), B(t)) of* (6.2 *are nonnegative for all* $t\geq0$*.*

**Proof**

It can be said that variable $\nu\left( t \right)\geq0$ for all $t\geq0$ if $\frac{d\nu}{dt}\geq0$ as $\nu\left( t \right)\to0$.

| $\left\{ \begin{aligned} \left. \frac{dS}{dt} \right\vert_{\xi\left( S \right)}=\mu\left( I+R \right)+\rho R\geq0, \\ \left. \frac{dI}{dt} \right\vert_{\xi\left( I \right)}=\beta_{1}BS\geq0, \\ \left. \frac{dR}{dt} \right\vert_{\xi\left( R \right)}=\eta I\geq0, \\ \left. \frac{d\hat{B}}{dt} \right\vert_{\xi\left( B \right)}=\epsilon I\geq0, \\ \end{aligned} \right.$ |  |
| --- | --- |

Where $\xi\left( \nu\right)= \{\nu\left( t \right)=0$ and $S,I,R,B \epsilon\mathfrak{R}_{0}^{+}\}$, and parameters $\mu,\rho,\beta,\eta,\epsilon\geq0$

We next prove that the human population remains constant such that $S\left( t \right)+I\left( t \right)+R\left( t \right)=N$ for all $t$

**Lemma 2.** *Given nonnegative initial population value* N, $\frac{dN}{dt}=0$ *for all* $t\leq0$*.*

**Proof:**

$$\frac{dN}{dt}=\frac{dS}{dt}+\frac{dI}{dt}+\frac{dR}{dt}$$

$\frac{dN}{dt}=\mu I+\mu R-\beta BS+\rho R+\beta BS-\mu I-\eta I+\eta I-\mu R-\rho R\equiv0$

Hence, if $S\left( t \right),I\left( t \right),R\left( t \right)\geq0$, and $S\left( t \right)+I\left( t \right)+R\left( t \right)=N$ for all values of $t$, then $S\left( t \right),I\left( t \right),R(t)$ must be bounded by N.

### Temperature Driven Model

Temperature influences cholera dynamics via the bacterial count. This was modelled by considering that temperature influences the growth rate and carrying capacity of bacteria with importance $\alpha$, as has been evidenced with other coastal bacteria [3]. $\alpha$ is a unitless parameter. This extended model is described by the following set of coupled ODEs.

| $\left\{ \begin{aligned} \frac{dS}{dt}=\mu\left( I+R \right)-\beta_{1}BS+\rho R \\ \frac{dI}{dt}=\beta_{1}BS-I\left( \mu+\eta\right) \\ \frac{dR}{dt}=\eta I-R\left( \mu+\rho\right) \\ \frac{dB}{dt}=\epsilon I+rT_{f}B\cdot\left( 1-\frac{B}{T_{f}C} \right) \end{aligned} \right.$ |  |
| --- | --- |
| $T_{f}(t)=\left( \frac{Temp}{Temp_{mean}} \right)^{\alpha}$ |  |

Where $T_{f}$ describes a ‘temperature factor’. $Temp_{mean}$ acts as a scaling factor and is equal to the mean daily temperature across the full timeseries dataset.

Positivity and Boundedness of Solutions

**Lemma 1.** *Given nonnegative initial conditions, the solutions (S(t), I(t), R(t), B(t)) of Eq.* *(*6.8) *are nonnegative for all* $t\geq0$*.*

**Proof**

It can be said that variable $\nu\left( t \right)\geq0$ for all $t\geq0$ if $\frac{d\nu}{dt}\geq0$ as $\nu\left( t \right)\to0$.

| $\left\{ \begin{aligned} \left. \frac{dS}{dt} \right\vert_{\xi\left( S \right)}=\mu\left( I+R \right)+\rho R\geq0, \\ \left. \frac{dI}{dt} \right\vert_{\xi\left( I \right)}=\beta_{1}BS\geq0, \\ \left. \frac{dR}{dt} \right\vert_{\xi\left( R \right)}=\eta I\geq0, \\ \left. \frac{d\hat{B}}{dt} \right\vert_{\xi\left( B \right)}=\epsilon I\geq0, \\ \end{aligned} \right.$ |  |
| --- | --- |

Where $\xi_{(\nu)}=\{\nu\left( t \right)=0$ and $S,I,R,B \epsilon\mathfrak{R}_{0}^{+}\}$, and parameters $\mu,\rho,\beta,\eta,\epsilon\geq0$

### Rainfall Model

This model contains an additional ‘$W$’ compartment, as proposed by Pascual et al [4] which tracks the volume of water per unit area present in the local aquatic environment, for example within urban ponds, canals and rivers. The modelled environment may be in one of two states: flooded or not flooded. The flooded state is defined by $W>W_{min}$, where $W_{min}$ describes the maximum water storage capacity per area of the modelled region with units $m^{3}/m^{2}$. $W$ is essentially a highly simplified flood model with a single input (Rainfall, $m$) and two outputs. The first is a continuous output $EW$ where $E$ represents a combination of evaporation and seepage (units = $day^{-1}$). When the system is ‘flooded’, a second, faster output is active representing drainage of flood waters at a rate of $DW_{flood}$ where $D$ represents drainage rate (units = $day^{-1})$ and $W_{flood}$ is the volume of water exceeding $W_{min}$.

The value of $W$ influences cholera dynamics in two ways

1. Bacterial Concentration: Infection rate is influenced by the *concentration* of bacteria in the environment, rather than merely bacterial numbers. This is introduced by swapping $B$ for $B/W$.
2. Flooding: When flooded, a flood factor, $F_{f}(t)$ (6.10) influences both the rate of excreted bacteria (due to breakdown of sanitation conditions) and infection rate (due to increased contact with contaminated environment via flooding). The flood factor is proportional to the W, and scaled by a static importance factor, $\omega$, which describes the importance of flooding such that:

| $F_{f}=1+\omega W_{flood}\left( t \right)$  $W_{flood}(t)=max(0,W-W_{min})$ |  |
| --- | --- |

This dual relationship with rainfall permits the complex relationship between cholera cases and rainfall witnessed in Kolkata where a potentially slight negative relationship was witnessed in the dry season, and a strong positive relationship was seen in the summer [5]. While in a ‘not flooded’ state, the system holds a negative relationship with rainfall, as this will reduce $W$ and therefore increase bacterial concentration. However, when the system enters the ‘flooded’ state, larger values of $W$ may lead to increases in both bacterial count and transmission rate. The resulting system of equations is given in below

| $\left\{ \begin{aligned} \frac{dS}{dt}=\mu\left( I+R \right)-\beta_{2}\frac{B}{W}F_{f}S+\rho R \\ \frac{dI}{dt}=\beta_{2}\frac{B}{W}F_{f}S-I\left( \mu+\eta\right) \\ \frac{dR}{dt}=\eta I-R\left( \mu+\rho\right) \\ \frac{dB}{dt}=\epsilon F_{f}I+rB\cdot\left( 1-\frac{B}{C} \right) \\ \frac{dW}{dt}=Rain\left( t \right)-DW_{flood}(t)-EW(t) \end{aligned} \right.$ |  |
| --- | --- |

Note that transmission is moderated by $\beta_{2}$ rather than by $\beta_{1}$ (as in the basic and temperature model). This is because while $\beta_{1}$ represents the effective contact rate per person per *bacteria* per day (units = $bacteria^{-1}day^{-1}$), $\beta_{2}$ represents the slightly different rate of contact per person per *concentration* per day (units = $m\cdot bacteria^{-1}day^{-1}$).

**Positivity and Boundedness of Solutions**

**Lemma 1.** *Given nonnegative initial conditions, the solutions (S(t), I(t), R(t), B(t), W(t)) of* (6.2)*are nonnegative for all* $t\geq0$*.*

**Proof**

It can be said that variable $\nu\left( t \right)\geq0$ for all $t\geq0$ if $\frac{d\nu}{dt}\geq0$ as $\nu\left( t \right)\to0$.

| $\left\{ \begin{aligned} \left. \frac{dS}{dt} \right\vert_{\xi\left( S \right)}=\mu\left( I+R \right)+\rho R\geq0, \\ \left. \frac{dI}{dt} \right\vert_{\xi\left( I \right)}=\beta_{2}\frac{B}{W}F_{f}S\geq0, \\ \left. \frac{dR}{dt} \right\vert_{\xi\left( R \right)}=\eta I\geq0, \\ \left. \frac{dB}{dt} \right\vert_{\xi\left( B \right)}=\epsilon I\geq0, \\ \left. \frac{dW}{dt} \right\vert_{\xi\left( W \right)}=Rain\geq0, \\ \end{aligned} \right.$ |  |
| --- | --- |

Where $\xi_{(\nu)}=\{\nu\left( t \right)=0$ and $S,I,R,B,W \epsilon\mathfrak{R}_{0}^{+}\}$ and parameters $\mu,\rho,\beta_{2},\eta,\epsilon,F_{f},Rain\geq0$

I next prove that the human population remains constant such that $S\left( t \right)+I\left( t \right)+R\left( t \right)=N$ for all $t$

**Lemma 2.** *Given nonnegative initial population value* N, $\frac{dN}{dt}=0$ *for all* $t\leq0$*.*

**Proof:**

$$\frac{dN}{dt}=\frac{dS}{dt}+\frac{dI}{dt}+\frac{dR}{dt}$$

$\frac{dN}{dt}=\mu I+\mu R-\beta_{2}\frac{B}{W}F_{f}S+\rho R+\beta_{2}\frac{B}{W}F_{f}S-\mu I-\eta I+\eta I-\mu R-\rho R\equiv0$

As in Section 6.3.2, if $S\left( t \right),I\left( t \right),R\left( t \right)\geq0$, and $S\left( t \right)+I\left( t \right)+R\left( t \right)=N$ for all values of $t$, then $S\left( t \right),I\left( t \right),R(t)$ must be bounded by N.

Dual Model

The final model combines the hypotheses of the Temperature and Rainfall models and consists of including the temperature factor described in the former into the latter model. Hence, the dual model can be described by the following set of coupled differential equations

| $\left\{ \begin{aligned} \frac{dS}{dt}=\mu\left( I+R \right)-\beta_{2}\frac{B}{W}F_{f}S+\rho R \\ \frac{dI}{dt}=\beta_{2}\frac{B}{W}F_{f}S-I\left( \mu+\eta\right) \\ \frac{dR}{dt}=\eta I-R\left( \mu+\rho\right) \\ \frac{dB}{dt}=\epsilon F_{f}I+rT_{f}B\cdot\left( 1-\frac{B}{T_{f}C} \right) \\ \frac{dW}{dt}=Rain\left( t \right)-DW_{flood}-EW \end{aligned} \right.$ |  |
| --- | --- |

Positivity and Boundedness of Solutions

**Lemma 1.** *Given nonnegative initial conditions, the solutions (S(t), I(t), R(t), B(t), W(t)) of* (6.2)*are nonnegative for all* $t\geq0$*.*

**Proof**

It can be said that variable $\nu\left( t \right)\geq0$ for all $t\geq0$ if $\frac{d\nu}{dt}\geq0$ as $\nu\left( t \right)\to0$.

| $\left\{ \begin{aligned} \left. \frac{dS}{dt} \right\vert_{\xi\left( S \right)}=\mu\left( I+R \right)+\rho R\geq0, \\ \left. \frac{dI}{dt} \right\vert_{\xi\left( I \right)}=\beta_{2}\frac{B}{W}F_{f}S\geq0, \\ \left. \frac{dR}{dt} \right\vert_{\xi\left( R \right)}=\eta I\geq0, \\ \left. \frac{dB}{dt} \right\vert_{\xi\left( B \right)}=\epsilon I\geq0, \\ \left. \frac{dW}{dt} \right\vert_{\xi\left( W \right)}=Rain\geq0, \\ \end{aligned} \right.$ |  |
| --- | --- |

Where $\xi_{(\nu)}=\{\nu\left( t \right)=0$ and $S,I,R,B,W \epsilon\mathfrak{R}_{0}^{+}\}$ and parameters $\mu,\rho,\beta_{2},\eta,\epsilon,F_{f},Rain\geq0$

Equations for human compartments $\left( \frac{dS}{dt},\frac{dI}{dt},\frac{dR}{dt} \right)$ are identical to the rainfall model. Therefore, proof for boundedness of human compartments for the dual model is equivalent.

### Dimensionality Reduction

In order to further simplify the number of unknown parameters in the models, and thereby simplify the calibration process and reduce computational expense, we employed a dimension reduction technique to streamline each system of ODEs. By rescaling the compartments in the models, this ensures that all compartments have the dimension of 1/time. This approach offers two distinct advantages. Firstly, the results obtained from the models become more interpretable, as many of the compartments initially contain extremely large numbers, and in certain cases, the scale may be unknown (e.g. water volume, number of bacteria). By reducing the dimensions, smaller numerical values are obtained that can be expressed relative to a fixed reference. This not only improves interpretability but also facilitates comparisons and provides a clearer understanding of the dynamics within the system. Secondly, reducing the dimensions contributes to computational efficiency during the parameter estimation step. By minimizing the number of unknown parameters and working with smaller numbers of similar scale, the improve performance of the MCMC algorithm can be improved [283] .

Here, human compartments were normalized by the population, $N$, such that $\hat{S}$,$\hat{I}$, and $\hat{R}$ represent proportions of the population and are equal to $S/N$, $I/N$, and $R/N$ respectively. The bacteria compartment $B$ is replaced by $\hat{B}$ which describes $B$ in terms of the carrying capacity $C$ such that $\hat{B}=\frac{B}{C}$. This transformation allows the elimination of C from the system of equations, simplifying the model structure and reducing the number of unknown parameters.

A reduced-dimension version of models 1-4 is given below.

**Model 1**

| $\left\{ \begin{aligned} \frac{d\hat{S}}{dt}=\mu\left( \hat{I}+\hat{R} \right)-\hat{\beta}\hat{B}\hat{S}+\rho\hat{R} \\ \frac{d\hat{I}}{dt}=\hat{\beta}\hat{B}\hat{S}-\hat{I}\left( \mu+\eta\right) \\ \frac{d\hat{R}}{dt}=\eta\hat{I}-\hat{R}\left( \mu+\rho\right) \\ \frac{d\hat{B}}{dt}=\hat{\epsilon}\hat{I}+r\hat{B}\left( 1-\hat{B} \right) \end{aligned} \right.$ |  |
| --- | --- |

**Model 2**

| $\left\{ \begin{aligned} \frac{d\hat{S}}{dt}=\mu\left( \hat{I}+\hat{R} \right)-\hat{\beta}\hat{B}\hat{S}+\rho\hat{R} \\ \frac{d\hat{I}}{dt}=\hat{\beta}\hat{B}\hat{S}-\hat{I}\left( \mu+\eta\right) \\ \frac{d\hat{R}}{dt}=\eta\hat{I}-\hat{R}\left( \mu+\rho\right) \\ \frac{d\hat{B}}{dt}=\hat{\epsilon}\hat{I}+rT_{f}\hat{B}\left( 1-\frac{\hat{B}}{Tf} \right) \end{aligned} \right.$ |  |
| --- | --- |

**Model 3**

| $\left\{ \begin{aligned} \frac{d\hat{S}}{dt}=\mu\left( \hat{I}+\hat{R} \right)-\hat{\beta}F_{f}\frac{\hat{B}}{\hat{W}}\hat{S}+\rho\hat{R} \\ \frac{d\hat{I}}{dt}=\hat{\beta}F_{f}\frac{\hat{B}}{\hat{W}}\hat{S}-\hat{I}\left( \mu+\eta\right) \\ \frac{d\hat{R}}{dt}=\eta\hat{I}-\hat{R}\left( \mu+\rho\right) \\ \frac{d\hat{B}}{dt}=\hat{\epsilon}F_{f}\hat{I}+r\hat{B}\left( 1-\hat{B} \right) \\ \frac{d\hat{W}}{\mathrm{dt}}=\hat{Rain}-D\hat{W_{flood}}-E\hat{W} \\ \end{aligned} \right.$ |  |
| --- | --- |

Where $\hat{W}=W/W_{min}$, $\hat{Rain}=Rain/W_{min}$

| $F_{f}\left( t \right)=1+\hat{\omega}\hat{W_{flood}}$  $W_{flood}=max(0,\hat{W}-1)$ |  |
| --- | --- |

**Model 4**

| $\left\{ \begin{aligned} \frac{d\hat{S}}{dt}=\mu\left( \hat{I}+\hat{R} \right)-\hat{\beta}F_{f}\frac{\hat{B}}{\hat{W}}\hat{S}+\rho\hat{R} \\ \frac{d\hat{I}}{dt}=\hat{\beta}F_{f}\frac{\hat{B}}{\hat{W}}\hat{S}-\hat{I}\left( \mu+\eta\right) \\ \frac{d\hat{R}}{dt}=\eta\hat{I}-\hat{R}\left( \mu+\rho\right) \\ \frac{d\hat{B}}{dt}=\hat{\epsilon}F_{f}\hat{I}+rT_{f}\hat{B}\left( 1-\frac{\hat{B}}{T_{f}} \right) \\ \frac{d\hat{W}}{\mathrm{dt}}=\hat{Rain}-D\hat{W_{flood}}-E\hat{W} \\ \end{aligned} \right.$ |  |
| --- | --- |

### Assumed Parameters

To reduce the unknowns in the models, two parameters which are well evidenced in the literature were assumed.

**Birth/death rate,** $\boldsymbol{\mu}$

Birth and death rate are assumed equal and take the value from the mean birth rate between 2011-2015. From 2011 this was 15.2 births annual births per 1000 population [279]. This converts to a value of $\mu=\frac{15.2}{1000\times365}=4.15\times{10}^{-5}$ births per person per day.

**Recovery Rate,**$\boldsymbol{\eta}$

A mean duration of infection of five days is assumed, a value commonly used in the literature [269,273,280,281][269,273,280,281], resulting in a recovery rate of 0.2 day^-1^. This is consistent with an early observational study which found that cholera patients experienced diarrhoea for a mean of 4.7 days [282] .

**References**

1. Vandermeer J. How Populations Grow: The Exponential and Logistic Equation. Nature Education Knowledge. 2010;3.

2. Yang C, Wang J. On the intrinsic dynamics of bacteria in waterborne infections. Math Biosci. 2018;296:71–81.

3. Huete-Stauffer TM, Arandia-Gorostidi N, Díaz-P ´ Erez L, Xos´ X, Anxelu X, Morán G, et al. Temperature dependences of growth rates and carrying capacities of marine bacteria depart from metabolic theoretical predictions. FEMS Microbiol Ecol. 2015;91:111.

4. Pascual M, Bouma MJ, Dobson AP. Cholera and climate: revisiting the quantitative evidence. Microbes Infect. 2002;4:237–45.

5. Shackleton D, Economou T, Memon F, Dutta S, Chen A, Kanungo S. Seasonality of Cholera in Kolkata and in the Influence of Climate. BMC Infect Dis. 2023.

6. Higdon D, Reese S, Moulton D, Vrugt J, Fox C. Posterior Exploration for Computationally Intensive Forward Models. In: Handbook of Markov Chain Monte Carlo. 1st edition. Chapman and Hall; 2011.

7. Government of West Bengal. Health on the March, 2015-2016. Kolkata; 2016.

8. Sun GQ, Xie JH, Huang SH, Jin Z, Li MT, Liu L. Transmission dynamics of cholera: Mathematical modeling and control strategies. Commun Nonlinear Sci Numer Simul. 2017;45:235–44.

9. Hartley DM, Morris JG, Smith DL. Hyperinfectivity: A Critical Element in the Ability of V. cholerae to Cause Epidemics? PLoS Med. 2005;3:e7.

10. Andrews JR, Basu S. Transmission dynamics and control of cholera in Haiti: an epidemic model. LANCET. 2011;377:1248–55.

11. Bertuzzo E, Mari L, Righetto L, Gatto M, Casagrandi R, Blokesch M, et al. Prediction of the spatial evolution and effects of control measures for the unfolding Haiti cholera outbreak. Geophys Res Lett. 2011;38:n/a-n/a.

12. Lindenbaum J, Greenough W, Benenson A, Oseasohn R, Rizvi S, Saad Anisa. NON-VIBRIO CHOLERA. Lancet. 1965.
