# Supplementary material for: Modelling the impact of climate on cholera: a case study of Kolkata
Source: Sci Rep. 2026 May 10;16:21346. doi: 10.1038/s41598-026-51415-z (PMC13346884; doi:10.1038/s41598-026-51415-z)
Supplement: Supplementary file 2 — Supplementary Information 2. [file 41598_2026_51415_MOESM2_ESM.docx]

**S3 – Parameter Estimation and Priors**

In each of the proposed models, there were between four and nine unknown parameters that must be inferred from the available data. Considering the available dataset comprises a relatively modest 252 autocorrelated datapoints, inferring this substantial number of parameters inevitably introduces uncertainty into our model outputs. It is therefore important to select a parameter estimation method which enables quantification of this uncertainty. The models are also non-linear and partially observed – the true state is unknown for any compartment at any time and as such inferences can only be made about the number of new infections at discrete time points indirectly via the number of reported cases. The parameter estimation technique therefore needs to be highly flexible. Finally, due to the high dimensionality of the potential parameter sample space, a computationally efficient approach is required to reasonably explore the parameter space within an acceptable time frame.

For these reasons, a Bayesian approach was taken due to its inherent ability to incorporate uncertainty of inputs and subjective prior beliefs. Specifically, we applied Markov Chain Monte Carlo (MCMC) estimation [1], a technique that allows us to generate samples from the posterior distribution, which in turn aids in making probabilistic statements about our model parameters.

The working principle of MCMC can be explained starting with Bayes formula (below) which details the relationship between the posterior probability of parameters, $\theta$ given the data, $y$ and the likelihood of the data given the parameters.

| $P\left( \theta\vert y \right)=\frac{P(y\vert\theta)\cdot P(\theta)}{P\left( y \right)}$ |  |
| --- | --- |

Here, $P(\theta|y)$ is the known as the *posterior distribution*, $P(y|\theta)$ the *likelihood function* and $P(\theta)$ the *prior distribution*. $P(y)$ is a normalizing constant which sums the numerator over all values of $\theta$ (i.e. $P\left( y \right)= \int P\left( y | \theta\right)P\left( \theta\right) d\theta$). This integral however, is intractable for many models including state space models such as those proposed in this paper, meaning that an analytical expression for the likelihood expression cannot be known. MCMC solves this problem by ignoring the normalizing constant and sampling directly from the posterior distribution by considering that $P\left( \theta| y \right)\propto P\left( y|\theta\right)\cdot P(\theta)$.

**Random-Walk Metropolis-Hastings Algorithm**

To perform MCMC estimation, the R package {*NIMBLE*} [2] was used which employs the Metropolis-Hastings algorithm to sample from the posterior distribution. This algorithm is described as follows. From some initial proposed (current) value of theta $(\theta_{j})$, a second proposed value ($\theta_{j+1})$ is selected from a normal distribution with mean equal to $\theta_{j}$ and standard deviation equal to a pre-selected proposal distribution $\sigma_{p}$.

| $\theta_{j+1}\mathcal{=N(}\theta_{j},\sigma_{p})$ |  |
| --- | --- |

The posterior for each $\theta$ is then evaluated as the product of the likelihood and prior as below

| $P\left( \theta_{j} \vert y \right)=P\left( y \vert\theta_{j} \right)\cdot P(\theta_{j})$ |  |
| --- | --- |

Next, the algorithm randomly chooses whether to accept the proposed $\theta$ according to an acceptance probability, $\alpha$, where $\alpha$ is calculated as the ratio of posteriors and can be no greater than 1.

| $\alpha=\min\left\{ 1, \frac{P\left( \theta_{j+1} \vert y \right)}{P\left( \theta_{j} \vert y \right)} \right\}$ |  |
| --- | --- |

The above steps are repeated for $n$ iterations, where the frequency distribution of $\theta_{1},\ldots,\theta_{n}$ represents the posterior distribution.

**Likelihood Function**

An essential part of MCMC is therefore the estimation of the marginal likelihood function, $P(y|\theta)$. To estimate this value across the entire dataset $y$ for a given value of $\theta_{j}$, the probability density function (PDF), $f$, is evaluated at each data point $y_{i}$. The overall likelihood is then given as the product of the individual likelihoods for each $N$ value $y_{i}$.

| $P\left( y \vert\theta\right)=\prod_{i=1}^{N} f(y_{i},\theta)$ |  |
| --- | --- |

In the context of this analysis, $y$ represents the cholera dataset, and hence $y_{i}$ represents the number of cholera cases recorded in Kolkata in month $i$. Because the proposed models do not directly track simulated monthly cholera cases, expected monthly cholera cases, $y.sim_{j}$, are instead estimated, where the $j$ subscript indicates a simulation run with parameters $\theta=\theta_{j}$.

$y.sim_{i,j}$ is calculated as the product of new infections in month $i$, $\hat{Z}_{i,j}N$, the proportion of infected individuals who will seek treatment at ID Hospital, $p$, and finally the proportion of diarrhoeal patients at ID Hospital who are randomly selected for bacteriological testing, $p_{test}$. As mentioned in section A2.1.2, around 6% of patients are tested, and therefore $p_{test}=0.06$.

| $y.sim_{i,j}=\hat{Z_{i,j}}N\cdot p\cdot p_{test}$ |
| --- |

In order to count new infections over time, an extra compartment, $\hat{Z}_{cum}(t)$, was introduced which describes the cumulative total number of infections which have occurred by time $t$, normalized by the population N, and is decoupled from the rest of the system. The differential equation governing the state of $\hat{Z}_{cum}$ is equal to the infection rate, where in the basic and temperature models:

| $\frac{d\hat{Z}_{cum}}{dt}=\hat{\beta_{1}}\hat{B}\hat{S}$ |  |
| --- | --- |
| And in the rainfall and dual models, $\hat{Z}_{cum}$ is described by: |  |
| $\frac{d\hat{Z}_{cum}}{dt}=\hat{\beta_{2}}F_{f}\frac{\hat{B}}{\hat{W}}\hat{S}$ |  |

New infections occurring in month $i$ are then calculated as $\hat{Z}_{i}$=$\hat{Z}_{cum,i}-\hat{Z}_{cum,i-1}$.

The data, $y$, is assumed to be sampled from a negative binomial distribution with mean, $\mu=y.sim_{i}$, and dispersion parameter, $size$. A negative binomial distribution was used due the count nature of the epidemiological data and potential presence of over-dispersion.

| $y_{i} \sim NegBinom\left( \mu=y.sim_{i},size \right)$ |
| --- |

An expression for the likelihood function $P(y|\theta_{j})$ is hence given by

| $P\left( y \vert\theta_{j} \right)=\prod_{i=1}^{N} dNegBinom(y_{i},y.sim_{i,j},size)$ |  |
| --- | --- |

Where $dNegBinom(y_{i},y.sim_{i,j},size)$ represents the probability density evaluated at point $y_{i}$ of a negative binomial distribution with mean $\mu=y.sim_{i,j}$ and dispersion parameter, $size$.

In this methodology, four concurrent ‘chains’ were run for each simulation, where a ‘chain’ refers to a sequence of iterations generated through the MCMC process. The benefit of operating multiple chains simultaneously is that it allows for cross-validation of their convergence. If all chains independently converge to the same posterior distribution, this acts as a reliable indicator of the robustness of the simulation [3]. For each chain, a total of $n=20,000$ MCMC iterations were run for each model. The first 18,000 iterations were discarded as ‘burn-in’ to ensure that samples recorded before the simulation reached convergence did not contribute to the overall posterior distribution for each parameter (See Supplementary Materials S4 for validation).

**Assumed Parameters**

**Birth/death rate** $\boldsymbol{\mu}$

Birth and death rate are assumed equal and take the value from the mean birth rate in Kolkata city between 2011-2015 of 15.2 births annual births per 1000 population [4]. This converts to a value of $\mu=\frac{15.2}{1000x365}=4.15x{10}^{-5}$ births per person per day

**Recovery Rate,** $\boldsymbol{\eta}$

A mean duration of infection of five days is assumed, a value commonly used in the literature [5–8], resulting in a recovery rate of 0.2 day^-1^.

**Informed Priors**

**Rate of Immunity loss,** $\boldsymbol{\rho}$

While many studies confirm that clinical cholera infection does confer some protection against future disease (e.g. [9–11]), estimates of the degree and duration of this protection vary widely, with duration estimates ranging from a few months to 9 years [12]. We represent this range of plausible values with a weakly informative prior $\rho\sim gamma(3,0.5)$

**Proportion of Hospitalized Infections, P**

Our prior assumptions on the proportion of cholera infections which result in the patient reporting to the Infectious Disease hospital are illustrated in Figure S3.1 According to a 2011 serological survey in Haiti [13], 78.7% of individuals infected with *Vibrio Cholerae* were asymptomatic, 6.0% experienced mild symptoms, 9.1% moderate and 6.2% severe. Due to lack of similar data in an endemic context, we extrapolate these results to the Kolkata context and suggest that ~50% of severe infections, ~30% of moderate infections, and ~5% of mild infections report to ID hospital, resulting in ~6.1% of total infections reporting to ID hospital. There is considerable uncertainty around these assumptions and therefore we represent this belief with a weakly informative prior described by $P\sim Beta(2,30)$


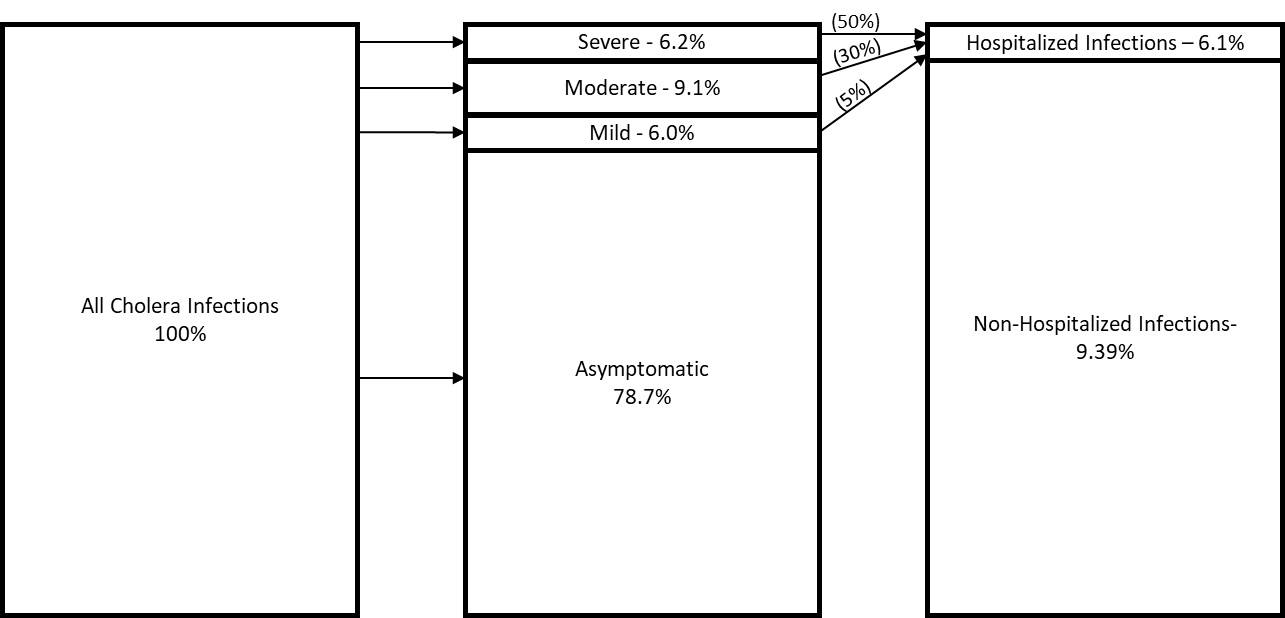


Figure S3.1 - Diagram of assumptions regarding P. Percentages in brackets are authors assumptions and not based on data

**Dispersion parameter, *size***

Due to the low proportion of infections reported as cases, we anticipate large amounts of noise in the data. We therefore provide a weakly informative prior with mean = 1, described by $size\sim Gamma(0.1,0.1)$. Small values of size imply the variance of the negative binomial distribution is larger than its mean, thus guarding against over-dispersion.

**W_min_**

The volume of water at which Kolkata becomes flooded is largely unknown. We assume it is on a similar scale as one month of monsoon rainfall (mean July rainfall over the observational period was around 0.3m). This is expressed as a weakly informed prior given by $W_{min}\sim Gamma(3,0.005)$

**Flood Drainage Rate, D**

The flood drainage rate descries the rate at which flood waters dissipate via natural or municipal drainage systems, as a proportion of its present volume. We are highly uncertain about this value but consider it likely to be less than 15% per day. We represent this with a weakly informative prior $D\sim Gamma(2,30)$

**Evaporation Rate, E**

The evaporation rate is expected to be much lower than the drainage rate and we consider this to be less than 5% of its present volume per day. We represent this with a weakly informative prior $D\sim Gamma(1,100)$

**Diffuse Priors**

No prior knowledge was identified regarding of the remaining parameters, and this was represented using uninformative priors. For parameters bound between [0,1] a flat beta prior (i.e. $\sim Beta(1,1)$) was used; else a uniform distribution was used, with upper and lower bounds selected to be as narrow as possible while still providing negligible influence on the posterior (see ‘Prior Predictive Checks’).

Table S3.1- Model Parameters

| *Model Parameters* | Description | Relation to original Parameters | Units | Prior Assumptions |
| --- | --- | --- | --- | --- |
| $\boldsymbol{\mu}$ | Birth/death rate | - | day^-1^ | 4.15 x 10^-5^ |
| $\boldsymbol{\eta}$ | Recovery rate | - | day^-1^ | 0.2 |
| $\boldsymbol{P}$ | Propotion of infections reported to ID hospital | - | Dimensionless | $\sim Beta(2,30)$ |
| ${\hat{\boldsymbol{\beta}}}_{\boldsymbol{1}}$ | Relative contact rate between humans and bacteria | $\beta C_{mean}$ | day^-1^ | $\sim Beta(1,1)$ |
| $\hat{\boldsymbol{\beta}_{\mathbf{2}}}$ | Relative contact rate between humans and infected water | $\frac{\beta C_{mean}}{W_{min}}$ | day^-1^ | $\sim Beta(1,1)$ |
| $\boldsymbol{\rho}$ | Rate of immunity loss | - | year^-1^ | $\sim Gamma(3,0.5)$ |
| $\hat{\boldsymbol{\epsilon}}$ | Relative excretion into environment rate | $\frac{\epsilon N}{C_{mean}}$ | day^-1^ | $\sim Unif\left( 0,1000 \right)$ |
| r | Growth rate of Vibrio Cholerae | - | day^-1^ | $\sim Beta(1,1)$ |
| $\boldsymbol{\alpha}$ | Temperature Factor | - | Dimensionless | $\sim Unif(0,50)$ |
| $\hat{\boldsymbol{\omega}}$ | Relative flood factor | $\omega W_{min}$ | Dimensionless | $\sim Unif(0,200)$ |
| $\boldsymbol{W}_{\boldsymbol{min}}$ | Water volume at which ‘flooding’ occurs | - | $m^{3}/m^{2}$ | $\sim Gamma(3,0.005)$ |
| $\boldsymbol{D}$ | Relative flood drainage rate | - | day^-1^ | $\sim Beta\left( 2,30 \right)$ |
| $\boldsymbol{E}$ | Evaporation rate | - | day^-1^ | $\sim Beta(1,100)$ |
| Size | Dispersion parameter | - | Dimensionless | $\sim Gamma(0.1,0.1)$ |

### **Initial Conditions**

In the proposed models, the initial conditions, i.e. the state of each compartment at time t=0, are not known. This presents a potential additional complexity in terms of the computation required if these conditions were treated as additional unknown parameters to be estimated. To avoid this, we first run each simulation for 10 years, the outputs of which are discarded, to allow the model reach endemic equilibrium. This approach has previously been successfully implemented in an environmentally driven cholera model by Bertuzzo et al. [14]. In this way, we assume that the model is not sensitive to the choice of initial conditions, and they may be selected arbitrarily. The selected initial conditions are given below.

| $\hat{S}_{0}=0.599$  $\hat{I}_{0}=0.001$  $\hat{R}_{0}=0.4$  $\hat{B}_{0}=1$  $\hat{W}_{0}=1$ |  |
| --- | --- |

Where subscript _0_ represents initial condition of variable.

**References**

1. Hastings WK. Monte Carlo Sampling Methods Using Markov Chains and Their Applications. Biometrika. 1970;57:97–109.

2. de Valpine P, Turek D, Paciorek CJ, Anderson-Bergman C, Lang DT, Bodik R. Programming With Models: Writing Statistical Algorithms for General Model Structures With NIMBLE. Journal of Computational and Graphical Statistics. 2017;26:403–13. https://doi.org/10.1080/10618600.2016.1172487/SUPPL_FILE/UCGS_A_1172487_SM7471.ZIP.

3. Roy V. Annual Review of Statistics and Its Application Convergence Diagnostics for Markov Chain Monte Carlo. 2019. https://doi.org/10.1146/annurev-statistics-031219.

4. Government of West Bengal. Health on the March, 2015-2016. Kolkata; 2016.

5. Hartley DM, Morris JG, Smith DL. Hyperinfectivity: A Critical Element in the Ability of V. cholerae to Cause Epidemics? PLoS Med. 2005;3:e7. https://doi.org/10.1371/journal.pmed.0030007.

6. Bertuzzo E, Mari L, Righetto L, Gatto M, Casagrandi R, Blokesch M, et al. Prediction of the spatial evolution and effects of control measures for the unfolding Haiti cholera outbreak. Geophys Res Lett. 2011;38:n/a-n/a. https://doi.org/10.1029/2011GL046823.

7. Sun GQ, Xie JH, Huang SH, Jin Z, Li MT, Liu L. Transmission dynamics of cholera: Mathematical modeling and control strategies. Commun Nonlinear Sci Numer Simul. 2017;45:235–44. https://doi.org/10.1016/j.cnsns.2016.10.007.

8. Andrews JR, Basu S. Transmission dynamics and control of cholera in Haiti: an epidemic model. LANCET. 2011;377:1248–55. https://doi.org/10.1016/S0140-6736(11)60273-0.

9. Ali M, Emch M, Park JK, Yunus M, Clemens JD. Natural Cholera Infection-Derived Immunity in an Endemic Setting. J Infect Dis. 2011;204:912–8. https://doi.org/10.1093/infdis/jir416.

10. Clemens JD, van Loon F, Sack DA, Rao MR, Ahmed F, Chakraborty J, et al. Biotype as determinant of natural immunising effect of cholera. The Lancet. 1991;337:883–4. https://doi.org/10.1016/0140-6736(91)90207-6.

11. Glass RI, Becker S, Huq I, Stoll BJ, Khan MU, MERSON MH, et al. Endemic cholera in rural Bangladesh, 1966-1980. Am J Epidemiol. 1982;116:959–70. https://doi.org/10.1093/oxfordjournals.aje.a113498.

12. Leung T, Matrajt L. Protection afforded by previous vibrio cholerae infection against subsequent disease and infection: A review. PLoS Negl Trop Dis. 2021;15:1–17. https://doi.org/10.1371/journal.pntd.0009383.

13. Jackson BR, Talkington DF, Pruckler JM, Fouché MDB, Lafosse E, Nygren B, et al. Seroepidemiologic survey of epidemic cholera in Haiti to assess spectrum of illness and risk factors for severe disease. American Journal of Tropical Medicine and Hygiene. 2013;89:654–64. https://doi.org/10.4269/ajtmh.13-0208.

14. Bertuzzo E, Mari L, Righetto L, Gatto M, Casagrandi R, Rodriguez-Iturbe I, et al. Hydroclimatology of dual-peak annual cholera incidence: Insights from a spatially explicit model. Geophys Res Lett. 2012;39. https://doi.org/10.1029/2011GL050723.
