# Supplementary material for: Modelling the impact of climate on cholera: a case study of Kolkata
Source: Sci Rep. 2026 May 10;16:21346. doi: 10.1038/s41598-026-51415-z (PMC13346884; doi:10.1038/s41598-026-51415-z)
Supplement: Supplementary file 3 — Supplementary Information 3. [file 41598_2026_51415_MOESM3_ESM.docx]

**S4 - Model Checks and Additional Results**

### **Prior Predictive Checks**

In order to verify the compatibility of the assumed prior knowledge with the simulated data, we performed a prior predictive check. The primary aim of this check was to ensure that the chosen priors led to sensible predictions. This check was accomplished by simulating data from the model using parameters drawn from the priors before any actual data were considered. In this analysis, we conducted 2000 simulations for each model. The parameters for these simulations were sampled directly from the prior distributions. These simulated data then provided a form of "predicted" or "expected" data distribution that reflects the prior knowledge and assumptions built into the model.

Figure S4.4 displays the central 60% of the simulated data representing the range between the first and fifth quantiles. The observed data lies well within the range of values generated by our simulations. This indicates that the observed data is not in conflict with the simulated data from our priors and reduces the chance that the choice of priors is biasing the model's estimates.


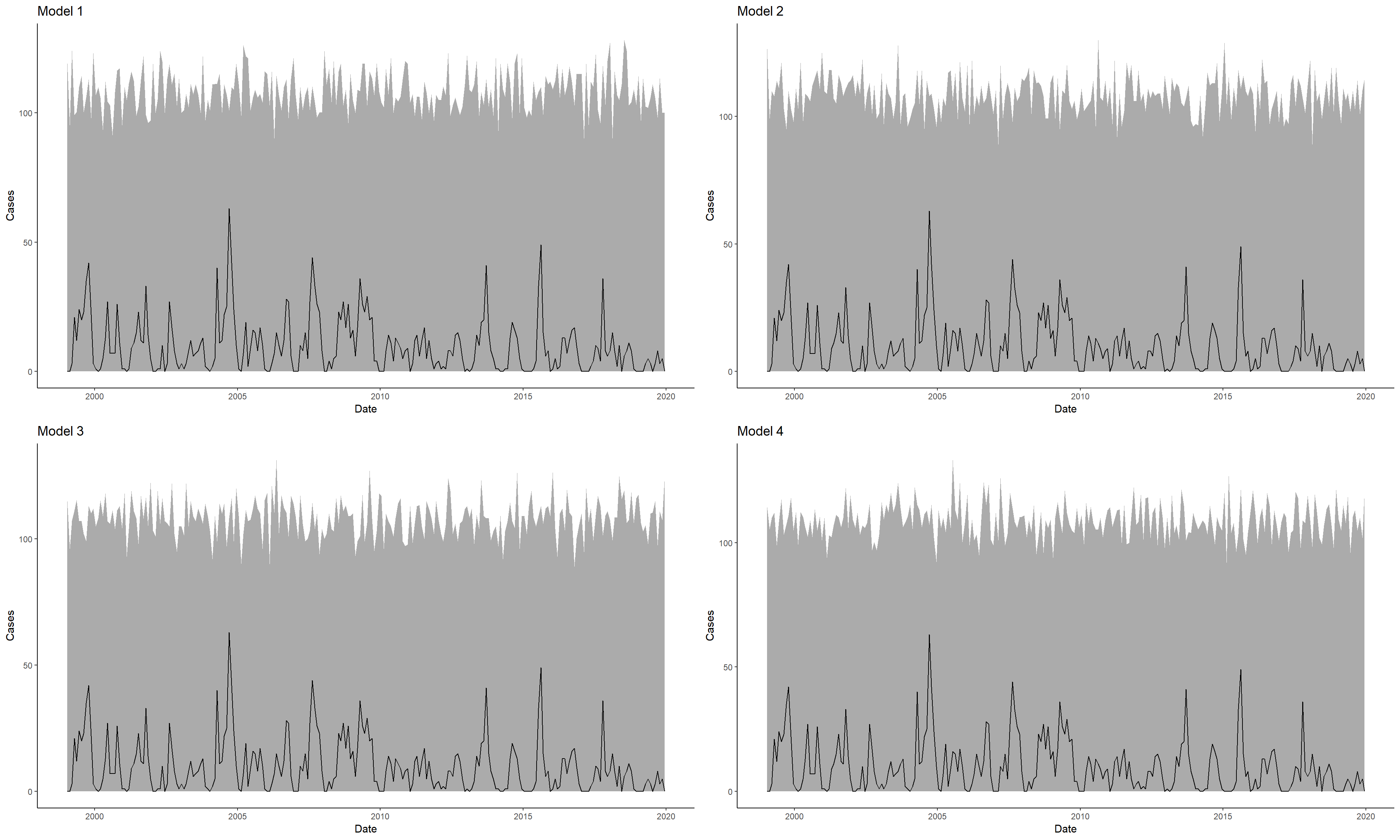


Figure S4.1 - Prior Predictive Checks: The grey area represents the range between the upper and lower quintiles of monthly cases estimated from 2000 simulations sampled from the prior distribution. The black line represents the observed monthly cases.

### **Sensitivity of Initial Conditions**

In the tested models, we assumed that the initial conditions of each model may be selected arbitrarily as it was assumed that 10 years is sufficient ‘warm up’ time to allow the model to reach endemic equilibrium. Sensitivity of this assumption was tested by running 10,000 simulations of each of the four models with initial conditions randomly sampled from uniform distributions as described in Table S4.1. To ensure that the sensitivity analysis was robust across a range of plausible parameter values, parameters were randomly sampled in each simulation from the posterior distribution. The influence of different initial conditions was measured by comparison with 10,000 simulations where initial conditions were fixed to those stated in Section Prior Distributions. To assess the similarity between the outputs of these two sets of simulations, we employed the Root Mean Squared Error (RMSE) as a measure of dissimilarity. The RMSE quantifies the average difference between corresponding outputs of the simulations, providing a comprehensive assessment of the overall model response.

The results of the sensitivity analysis are depicted in Figure S4.2 which illustrates the RMSE between the outputs associated with the selected initial conditions and randomly sampled initial conditions for each model. It is evident from columns 1 and 3 of Figure S4.2 that the model is most sensitive to assumptions about $\hat{S}_{0}$ and $\hat{R}_{0}$ with selected values closest to model assumptions (i.e. $\hat{S}_{0}$=0.599, $\hat{R}_{0}$=0.4) displaying greatest similarity. Outputs associated with values of $\hat{I}_{0}$, $\hat{B}_{0}$, and $\hat{W}_{0}$ do not appear to hold any relationship with its similarity to outputs of arbitrarily selected initial conditions, indicating complete insensitivity. Overall, for each model, the mean RMSE was < 0.2, hence a 10-year ‘warm up’ time was considered sufficient.

Table S4.1 - Initial condition sampling distributions for sensitivity analysis. $\boldsymbol{Unif(a,b)}$ represents a uniform distribution with lower and upper limits a and b respectively.

| Init. Cond. | ${\hat{\boldsymbol{S}}}_{\boldsymbol{0}}$ | ${\hat{\boldsymbol{I}}}_{\boldsymbol{0}}$ | ${\hat{\boldsymbol{R}}}_{\boldsymbol{0}}$ | ${\hat{\boldsymbol{B}}}_{\boldsymbol{0}}$ | ${\hat{\boldsymbol{W}}}_{\boldsymbol{0}}$ |
| --- | --- | --- | --- | --- | --- |
| Sample Distribution | $1-I0-B0$ | $Unif(0,0.1)$ | $Unif(0,\left( 1-I0 \right))$ | $Unif(0,1)$ | $Unif(0,1)$ |


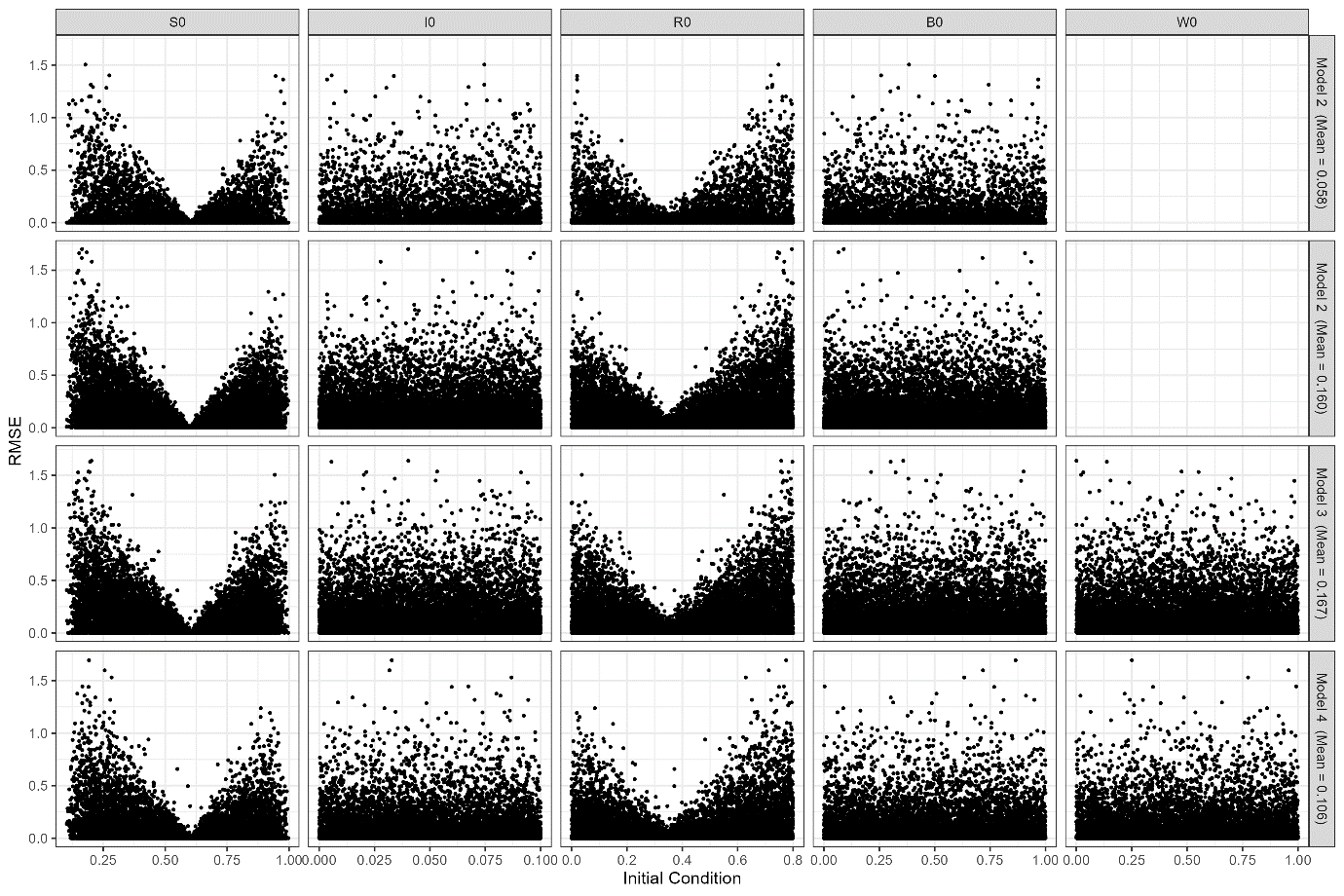


Figure S4.2- Sensitivity analysis of initial conditions to model outputs. RMSE between simulations with randomly sampled initial conditions, and initial conditions used in the parameter estimation for 10,000 simulations for each model. Mean RMSE score for each model is shown on y label on the right of the figure.

### **Model Convergence**

A key measure of reliability in Markov Chain Monte Carlo (MCMC) simulations is convergence. This refers to the point at which a chain stabilizes to a consistent distribution that closely approximates the model parameters' true posterior distribution. Model convergence indicates that MCMC estimates of model parameters are consistent, unbiased, and reliable.

The traceplots for each of the model simulations are shown in Figure S4.3-6 **.** For each model, 20,000 MCMC iterations were performed per chain, with the first 18,000 iterations discarded as burn-in to remove dependence on initial parameter values. Model convergence was inferred by considering the potential scale reduction factor of the remaining 2,000 iterations, commonly known as R-hat. This MCMC diagnostic quantifies the extent to which the variance between chains' means exceeds the expected value under identical distribution conditions. A value below 1.1 for each parameter is considered to indicate acceptable convergence (Gabry and Goodrich, 2023) and this is true for all parameters in all models except for $\hat{\beta_{2}}$ in the Rainfall Model which has an R-hat value of 1.17. This indicates that the posterior distributions are no longer influenced by initial parameter values and hence a burn-in of 18,000 was acceptable.

The prior and posterior distributions of MCMC simulations for all models are shown for each chain in Figure S4.7-10**.** The strong agreement between chains for all parameters is a further indication of good model convergence. For certain parameters such as $\hat{\beta}$**,** $\alpha$ the posterior distribution suggests a much narrower range of possible values compared to their respective priors. This indicates that the calibration process has added considerable information to these unknowns and our understanding of the ‘true’ value of these parameters is much improved. However, for other parameters such as and $W_{min}$ the posterior distributions are only marginally narrower than their respective priors. This indicates that there is a large range of possible parameter values which can reasonably reproduce the data, and as such it is clear that considerable uncertainty remains in the model.


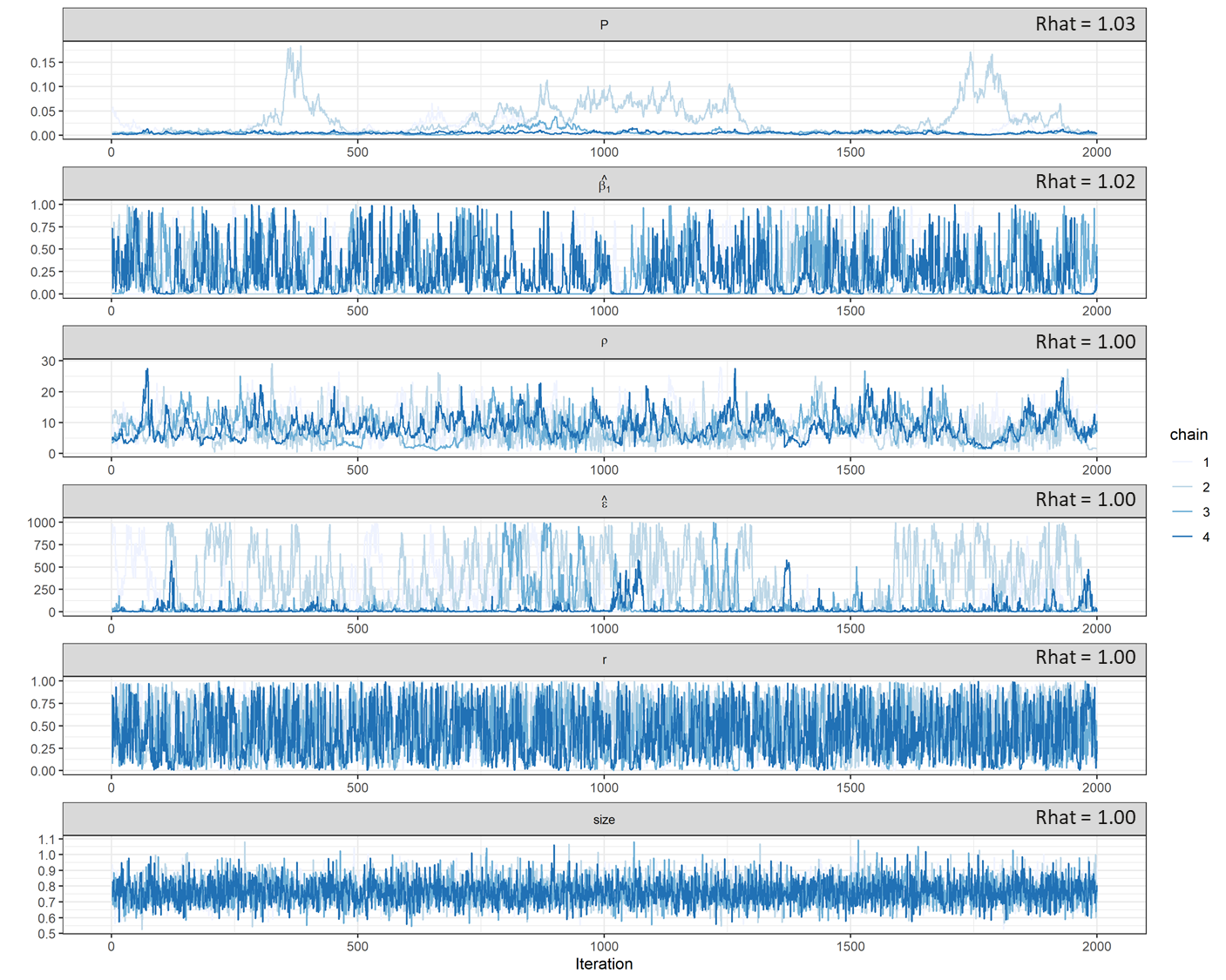


Figure S4.3- Basic Model MCMC traceplot. First 18000 iterations are omitted for clarity. Rhat for each parameter is given in top-right of each plot.


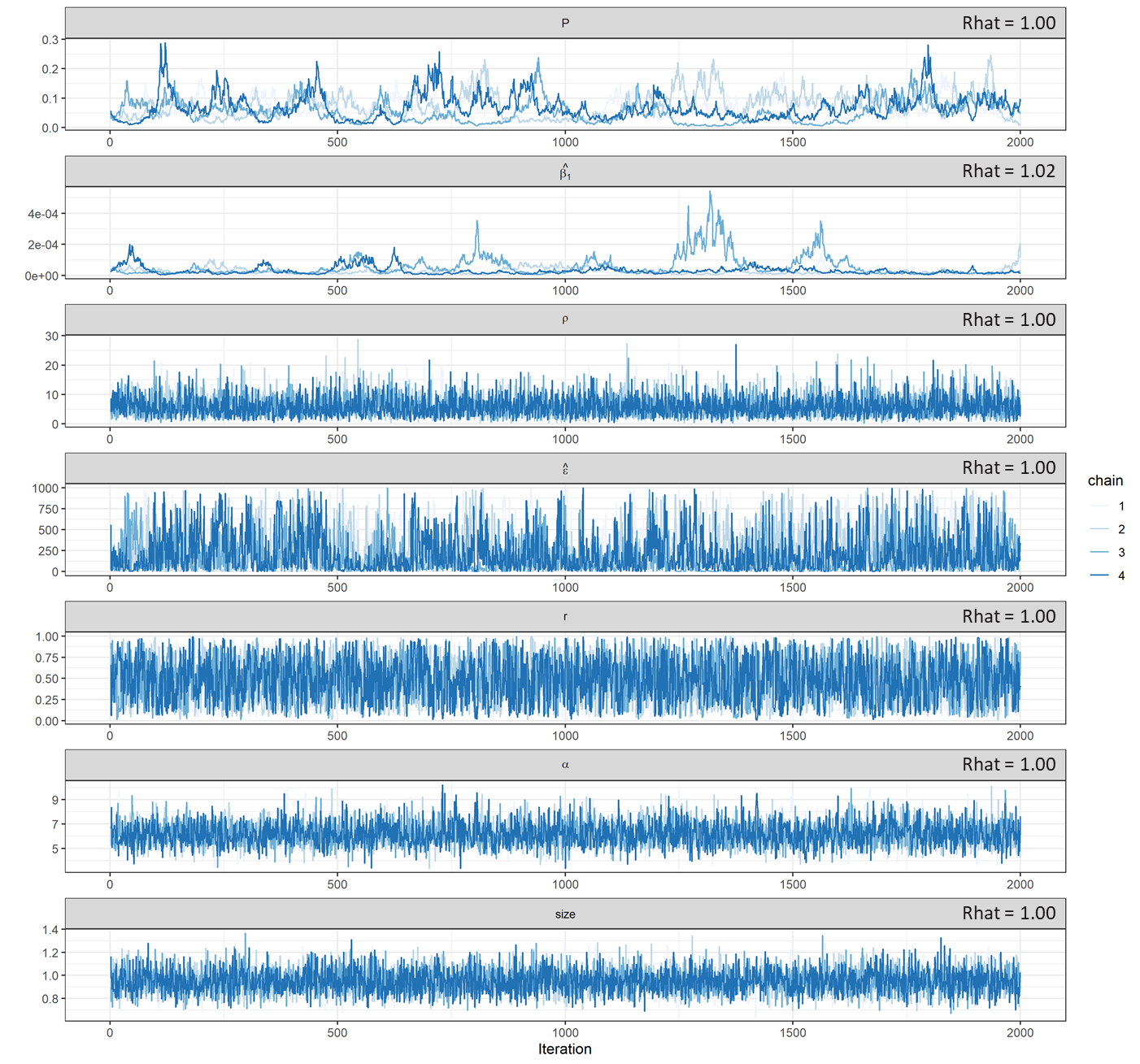


Figure S4.4- Temperature MCMC traceplot. First 18000 iterations are omitted for clarity. Rhat for each parameter is given in top-right of each plot.


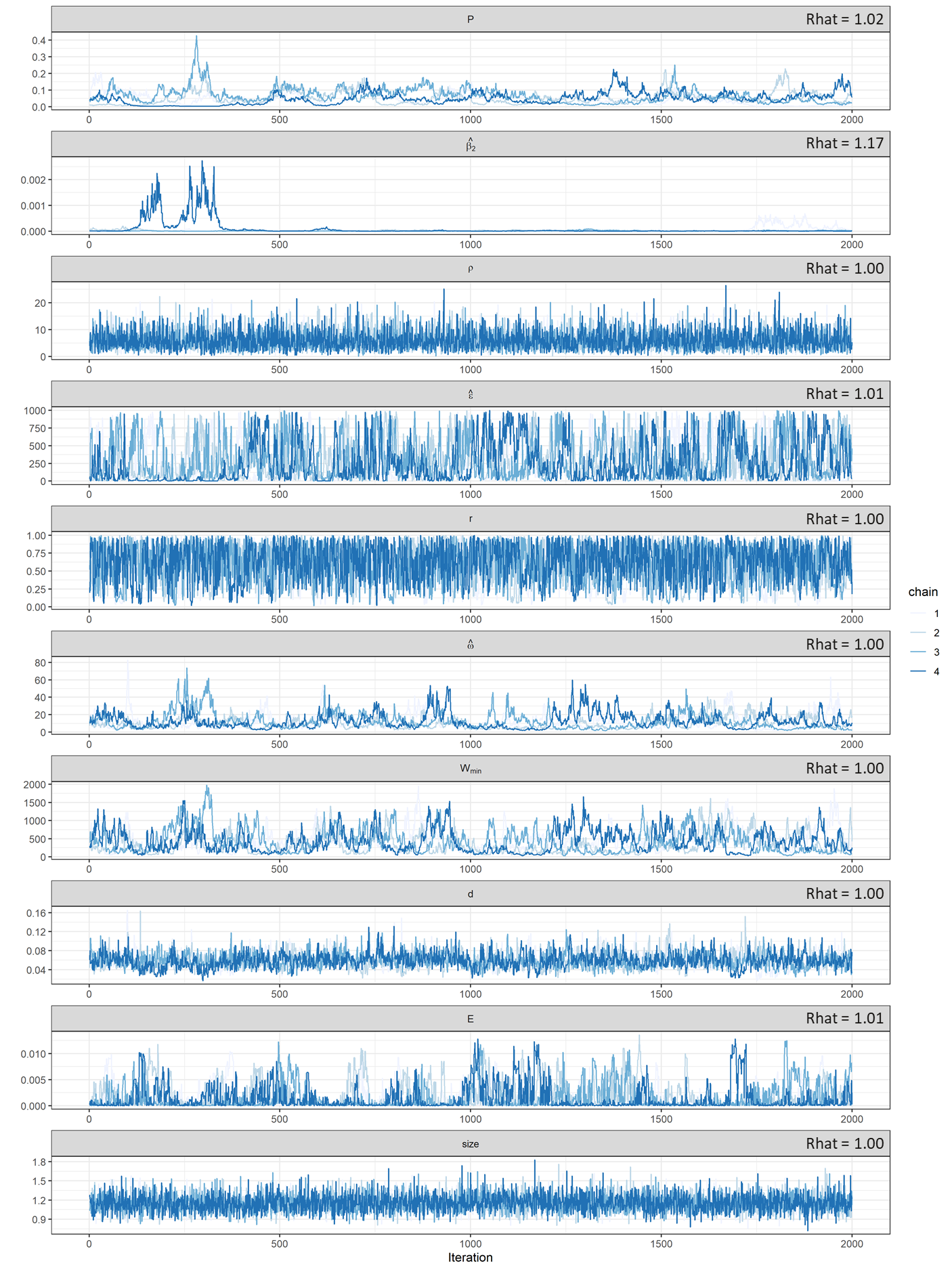


Figure S4.5– Rainfall Model MCMC traceplot. First 18000 iterations are omitted for clarity. Rhat for each parameter is given in top-right of each plot.


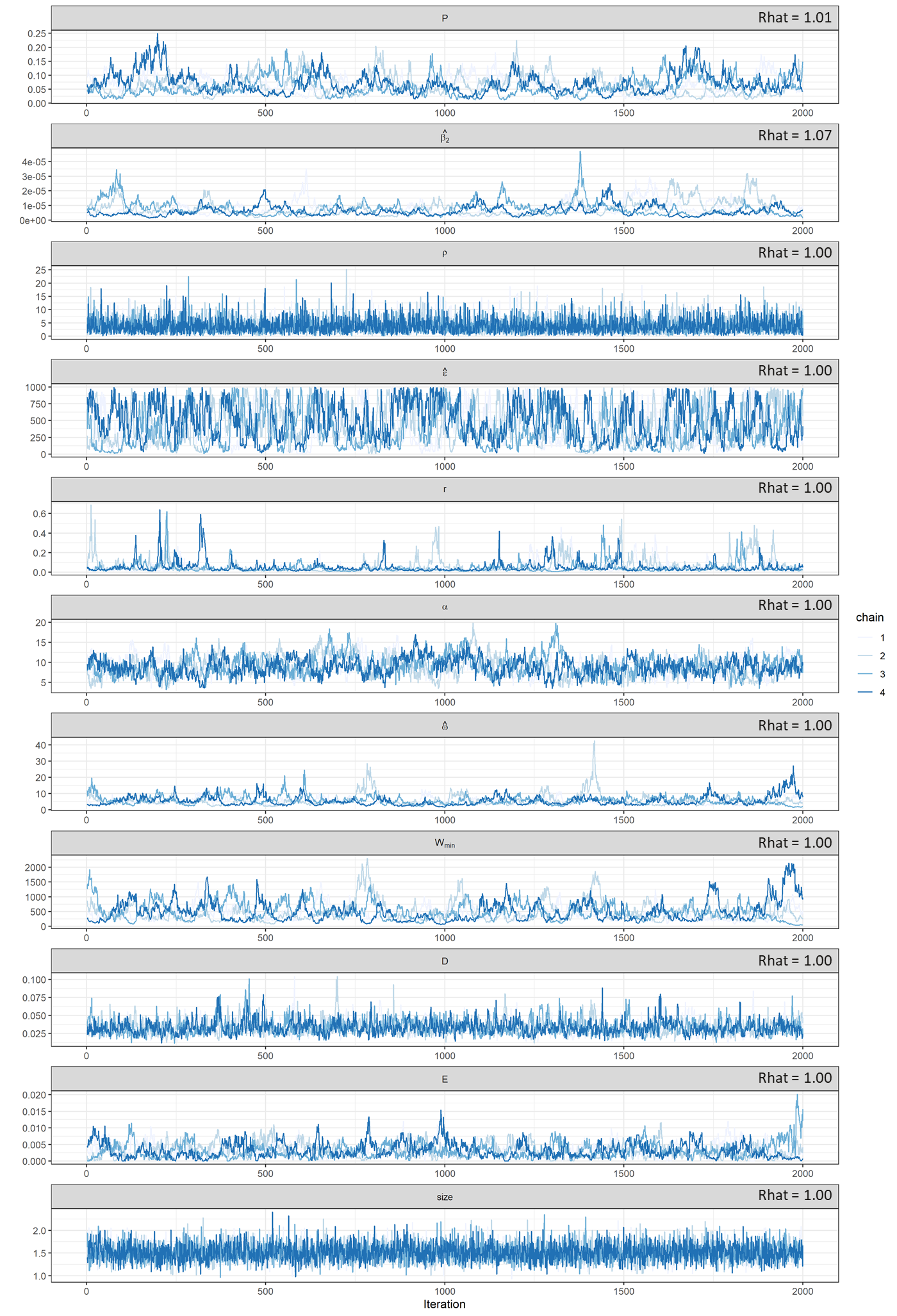


Figure S4.6- Dual Model MCMC traceplot. First 18000 iterations are omitted for clarity. Rhat for each parameter is given in top-right of each plot.


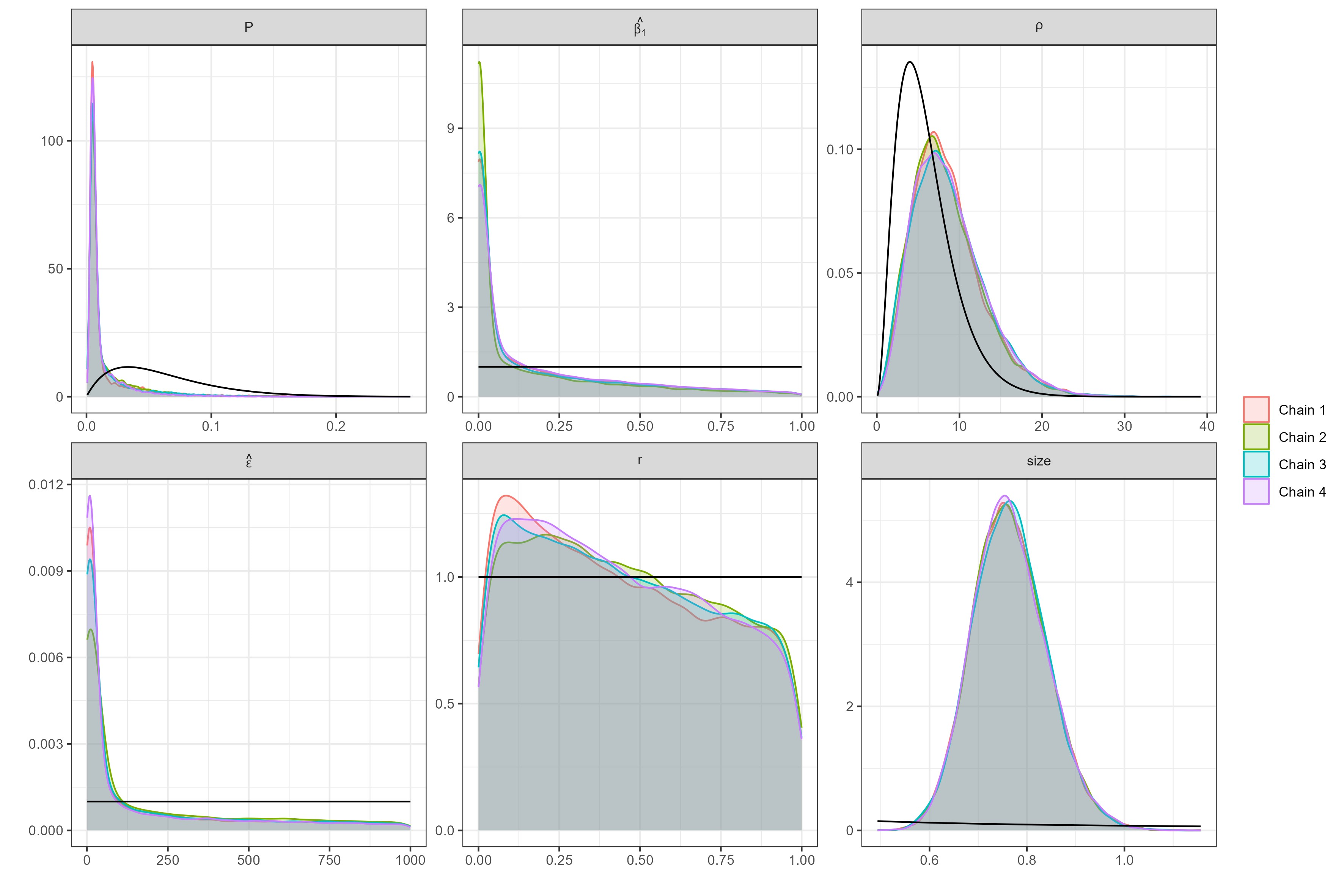


Figure S4.7– Prior and posterior density plot for Basic Model parameters. Posterior density is shaded by chain. Black line denotes prior probability assumption.


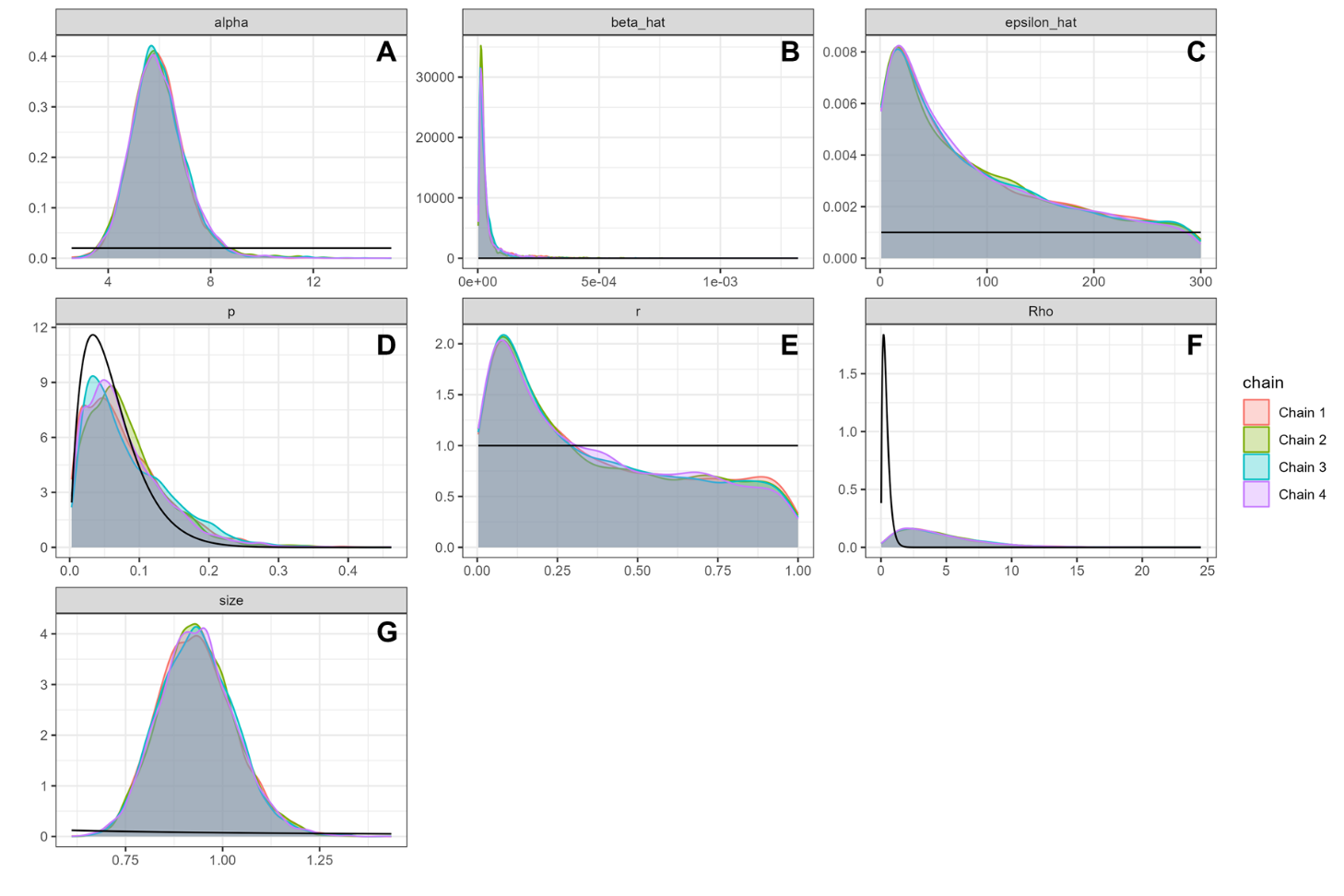


Figure S4.8- Prior and posterior density plot for Temperature Model parameters MCMC simulation. Posterior density is shaded by chain. Black line denotes prior probability assumption.


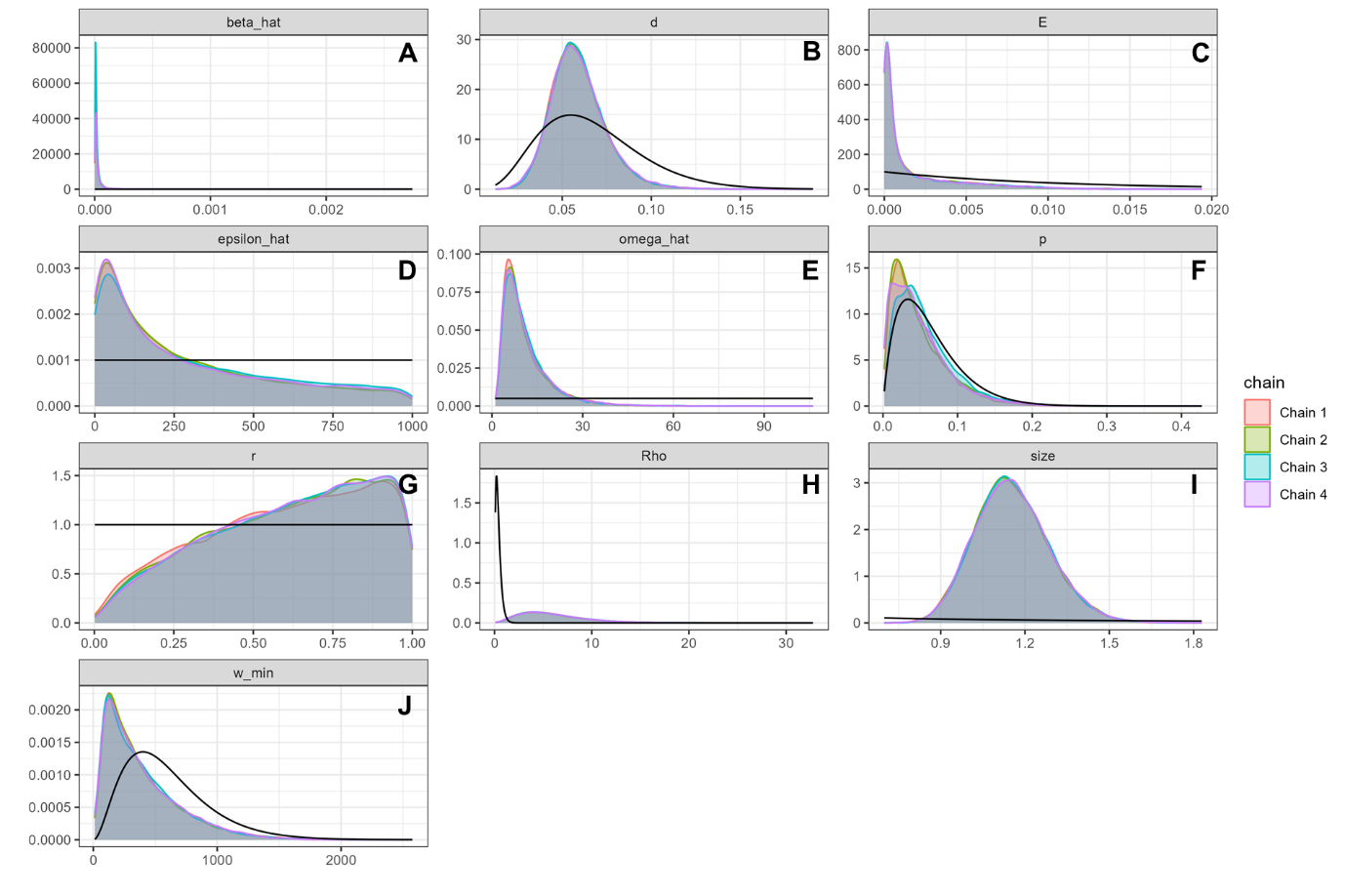


Figure S4.9- Prior and posterior density plot for Rainfall Model parameters. Posterior density is shaded by chain. Black line denotes prior probability assumption.


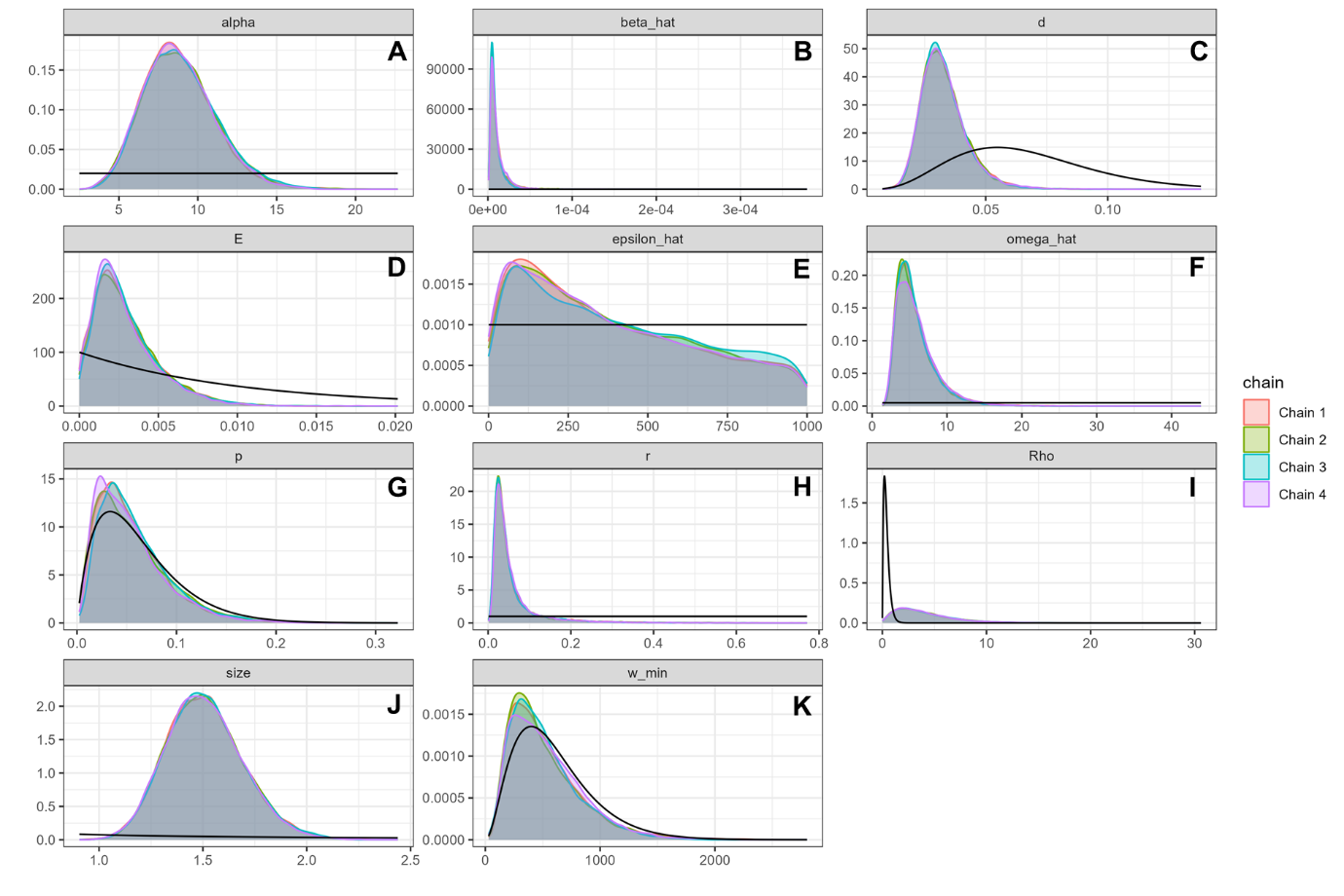


Figure S4.10- Prior and posterior density plot for Dual Model parameters. Posterior density is shaded by chain. Black line denotes prior probability assumption.

**Additional Results**

Table S4.2 - Relative increase in mean monthly cholera infections projected for 2080–2099 under each SSP scenario compared with the reference period (1999–2019). Values represent multiplicative changes in simulated mean monthly cases (e.g. 2.0 indicates a 100% increase relative to the reference period).

|  | Jan | Feb | Mar | Apr | May | Jun | Jul | Aug | Sep | Oct | Nov | Dec |
| --- | --- | --- | --- | --- | --- | --- | --- | --- | --- | --- | --- | --- |
| SSP1-2.6 | 1.62 | 1.36 | 1.23 | 1.5 | 1.92 | 2.32 | 2.1 | 1.97 | 1.82 | 1.58 | 1.63 | 1.71 |
| SSP2-4.5 | 1.61 | 1.47 | 1.38 | 1.67 | 2.14 | 2.6 | 2.39 | 2.00 | 1.82 | 1.56 | 1.55 | 1.66 |
| SSP5-8.5 | 2.02 | 1.98 | 2.28 | 2.43 | 2.79 | 3.52 | 3.11 | 2.51 | 2.3 | 2.02 | 2.19 | 2.28 |
