# Supplementary material for: Modelling the impact of climate on cholera: a case study of Kolkata
Source: Sci Rep. 2026 May 10;16:21346. doi: 10.1038/s41598-026-51415-z (PMC13346884; doi:10.1038/s41598-026-51415-z)
Supplement: Supplementary file 4 — Supplementary Information 4. [file 41598_2026_51415_MOESM4_ESM.docx]

## **S2 - Climate Data and Downscaling**

**Reference Climate Data (1984-2019)**

Daily mean temperature (°C) and total precipitation (mm/day) were inferred by extracting daily estimations provided by the CRU Japanese Reanalysis (CRU JRA – available at <https://catalogue.ceda.ac.uk/>) [1]. Each dataset was available at a 0.5x0.5 degree spatial resolution and the cell with centroid longitude = 88.25°E, latitude = 22.75°N was chosen since it covered the majority of the KMC area (BB1 in Figure S2.1).


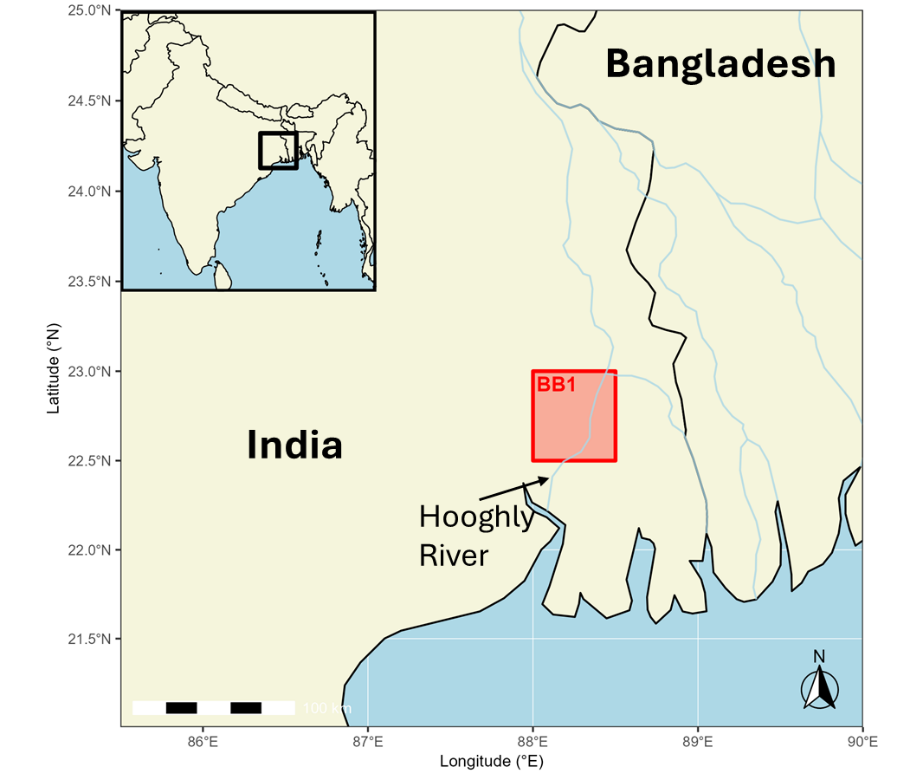


Figure S2.1 - Map of the case study area. The red shaded cell (BB1) indicates the region from which climate data were extracted, centred at 88.25°E, 22.75°N with a spatial resolution of 0.5° × 0.5°. The inset shows the location of the study area within the broader Indian subcontinent. Map created by the authors using R (v4.3.1) with the packages ggplot2, sf, and rnaturalearth. Country boundaries and river data sourced from Natural Earth (public domain; www.naturalearthdata.com).

**Climate Projections (2070-2099)**

Because GCMs all make unique simplifying assumptions about climate processes, there remains considerable uncertainty surrounding their projections. To incorporate and quantify this uncertainty, projections from 10 independent climate models from different institutions were selected to produce a more holistic view of plausible climate projections. These are outlined in Table S2.1.

Table S2.1– List of raw GCM outputs used in the study

| GCM | Institution, Country | Spatial Resolution |
| --- | --- | --- |
| ACCESS-CM2  [2] | Commonwealth Scientific and Industrial Research Organisation (CSIRO), Australia | 1.25° x 1.88° |
| AWI-CM  [3] | Alfred Wegener Institute (AWI), Germany | 0.94° x 0.94° |
| CMCC-ESM2  [4] | Centro Euro-Mediterraneo Sui Cambiomenti Climatici (CMCC), Italy | 0.94° x 1.25° |
| IITM-ESM  [5] | Centre for Climate Change Research (CCCR), India | 1.90° x 1.88° |
| MIROC6  [6] | Japan Agency for Marine-Earth Science and Technology (JAMSTEC), Japan | 1.40° x 1.41° |
| INM-CM5-0  [7] | Institute for Numerical Mathematics (INM), Russia | 1.5° x 2° |
| NorESM2-LM  [8] | Norwegian Climate Centre (NCC) | 1.9° x 2.5° |
| CESM2  [9] | National Centre for Atmospheric Research (NCAR), USA | 0.94° x 1.25° |
| IPSL-CM6A-LR  [10] | Institute Pierre-Simon Laplace (IPSL), France | 1.2° x 2.5° |

**Bias Correction and Downscaling**

Each of the climate projections used in the this study were first corrected for systematic biases using the Change Factor Methodology (CFM) which is commonly used in impact studies [11]. While other, more sophisticated bias-correction methodologies exist (e.g. trend-preserving quantile mapping [12]), which are better able to account for changes in extreme values of rainfall, CFM was considered to be acceptable for this analysis as the model takes the rolling mean of daily rainfall, and thus extreme values are not relevant. CFM also has the additional advantage of preserving the (generally higher) spatial resolution of the reference data.

Specifically, the CFM methodology outlined by Tabor and Williams [13] was used. The basic assumption made is that, while the absolute climate projection is likely biased, the modelled climate anomaly, (i.e. the difference between the future model output and a reference model output) is unbiased. Therefore, bias-corrected absolute projected values of temperature and rainfall for each GCM and SSP output can be obtained by calculating the respective modelled anomaly outputs and combining the observed data from a reference period. This method automatically downscales the dataset as the anomaly is assumed to be consistent over a large geographic area. A flow chart of the method is provided in Figure S2.2


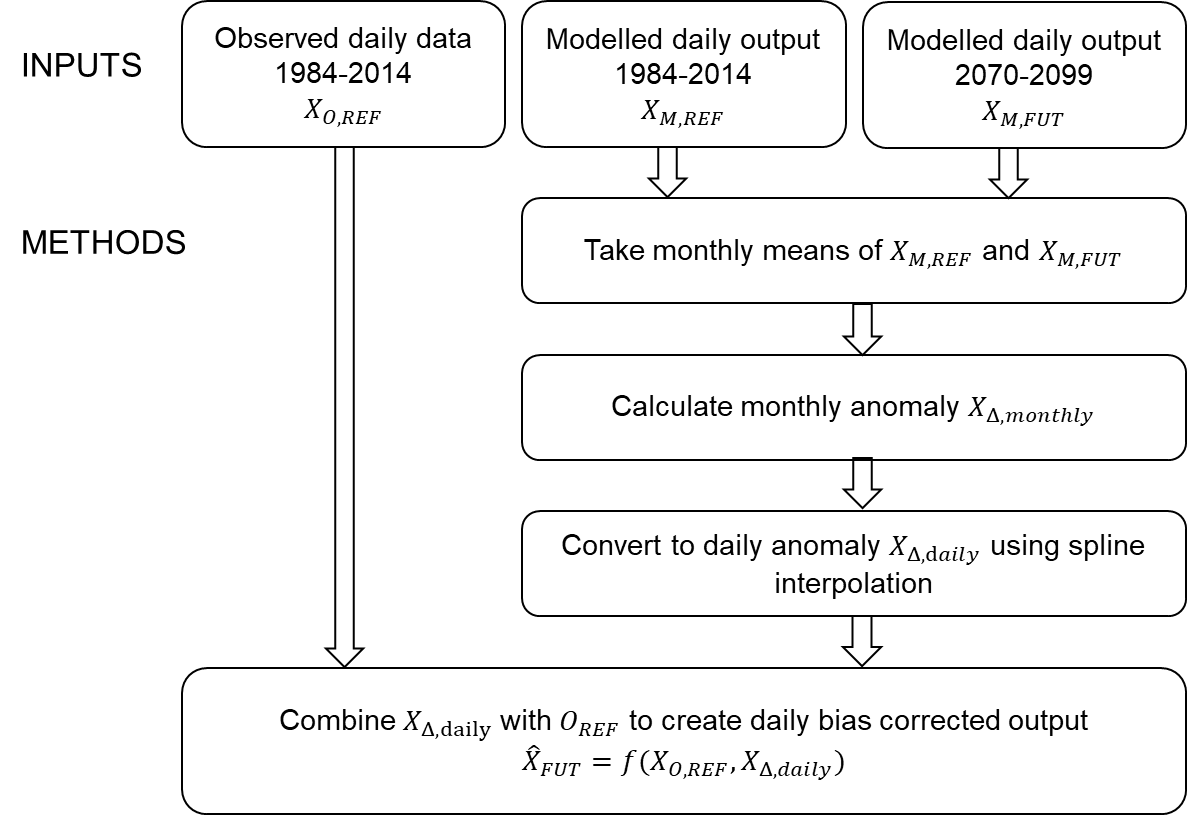


Figure S2.2– Flow chart of CFM methodology, adapted from [13]. Where $\boldsymbol{X}$ represents the climate variable. Subscripts O,REF denotes observed variable during the reference period; M,REF the modelled variable during the reference period; M,FUT the projected modelled variable; and $\boldsymbol{\Delta}$ the variable anomaly. ${\hat{\boldsymbol{X}}}_{\boldsymbol{FUT}}$ refers to the bias-corrected projected variable. This methodology was repeated for each GCM model and for each SSP output produced by each model.

First, the anomaly means are calculated for each month of the year in order to retain seasonal differences in anomalies values. For temperature, this is estimated as the monthly projected climatological average for the future period 2070-2099 minus the monthly modelled climatological normal for the reference period 1984-2014. The anomaly is estimated individually for each GCM.

| $Temp_{\Delta,month}=Temp_{M,FUT,month}-Temp_{M,REF,month}$ |  |
| --- | --- |

Where $Temp_{\Delta,month}$ is the temperature anomaly in each month of the year, $Temp_{M,FUT,month}$ is the mean GCM modelled temperature in each month of the year for the future period, and $Temp_{M,REF,month}$ is the mean GCM modelled temperature in each month of the year for the reference period.

Monthly rainfall anomaly, $Rainfall_{\Delta,month}$ is calculated as the relative difference between the mean monthly rainfall over future and reference periods as in [14].

| $Rainfall_{\Delta,month}=\frac{Rainfall_{M,FUT,monthly}}{Rainfall_{M,REF,monthly}}$ |  |
| --- | --- |

Where ${Rainfall}_{\Delta,month}$ is the rainfall anomaly in each month of the year, ${Rainfall}_{M,FUT,month}$ is the mean GCM modelled rainfall in each month of the year for the future period, and ${Rainfall}_{M,REF,month}$ is the mean GCM modelled rainfall in each month of the year for the reference period.

To avoid sharp unrealistic changes in month-to-month anomalies, anomalies were smoothed to daily values using cubic spline interpolation. The bias corrected future projection was then obtained by combining the estimated daily anomaly with the observed values from the reference period.

| $\hat{Temp}_{FUT}=Temp_{O,REF}+Temp_{\Delta,daily}$ |  |
| --- | --- |
| $\hat{Rainfall}_{FUT}=Rainfall_{O,REF}\times Rainfall_{\Delta,daily}$ |  |

Where $\hat{Temp}_{FUT}$ and $\hat{Rainfall}_{FUT}$ are the bias-corrected future projections for rainfall and temperature respectively, while $Temp_{O,REF}$ and $Rainfall_{O,REF}$ represent the observed values due the reference period for temperature and rainfall respectively.

**Climate Projection Outputs**

The results of the bias-correction and downscaling for projected Kolkata temperatures in 2070-2099 are given in Figure S2.3. A rise in mean temperatures of 1.30°C, 1.94°C, and 3.62°C was estimated for scenarios SSP1-2.6, SSP2-4.5 and SSP5-8.5 respectively. These increases in temperature did not appear to change notably between seasons. The range of model outputs produced by different GCMs is of a similar scale at 1.30°C, 1.76°C and 2.70°C, indicating moderate agreement between models and thus moderate confidence can be attributed in model predictions of temperature, with greatest confidence in projections with the lowest radiative forcing.


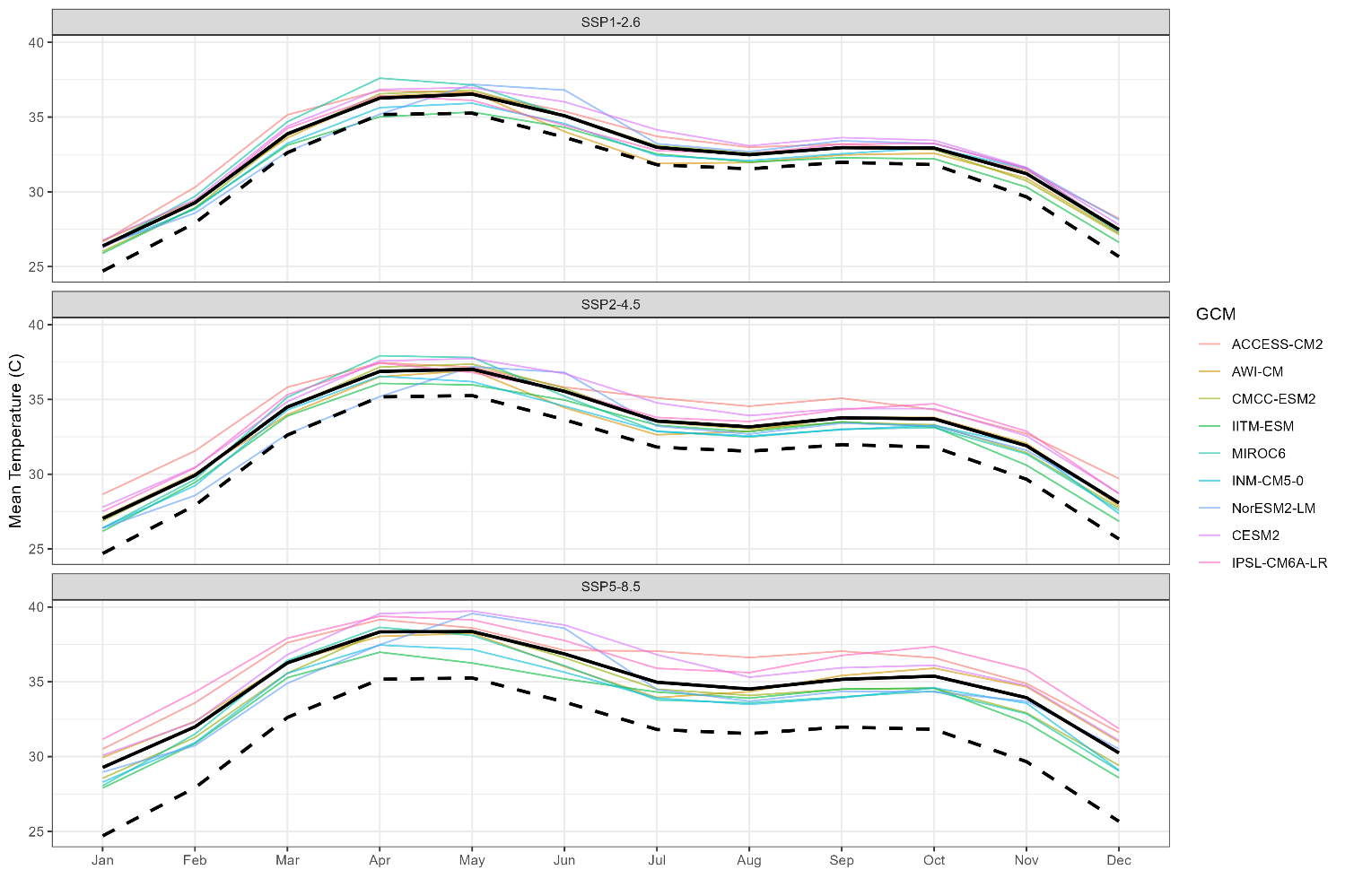


Figure S2.3 - Bias-corrected temperature projections in Kolkata. Coloured lines give the bias corrected average temperature for each GCM under forcing scenarios SSP1-2.6, SSP2-4.5, and SSP5-8.5 during the future period 2070-2099. Solid black line shows the mean average of all GCM outputs for each scenario. Dashed black represents the average temperature according to the observed reference dataset 1984-2014. Monthly average values are shown to emphasize general model differences.

Projections for Kolkata rainfall in the future period are given in Figure S2.4. In all SSP scenarios, there are strong seasonal differences in projected rainfall anomalies. Between December and April, there is strong agreement across all GCMs that the mean rainfall in this period will be highly similar in all three future SSP scenarios and the reference period. From May-July, the projected change in mean rainfall remains slight, though with much greater variability. While GCMs generally agree a slight decrease in the rainfall under SSP1-2.6, under SSP2-4.5 and SSP5-8.5 model outputs range from mean increase of 36% and 46% respectively (both resulting from the ACCESS-CM2 model) to a mean decrease of 64% and 35% respectively (resulting from NorESM2-LM and CESM2 models). Perhaps the most interesting finding, however, comes during the months August-November. All GCMs considered in the study and across all SSP scenarios (with the single exception of NorESM2-LM under SSP2-4.5), have projected a general increase in rainfall during this period and also an extension of the monsoon of around a month later in the year. The precise average magnitude and seasonal distribution of the projected future monsoon, however, varies significantly between models; most notably with regards to the AWI-CM model which projects an increase in September rainfall of over 350% in all SSP scenarios. As this is significantly greater than any other model projects, this output was considered an outlier and removed from further analysis.


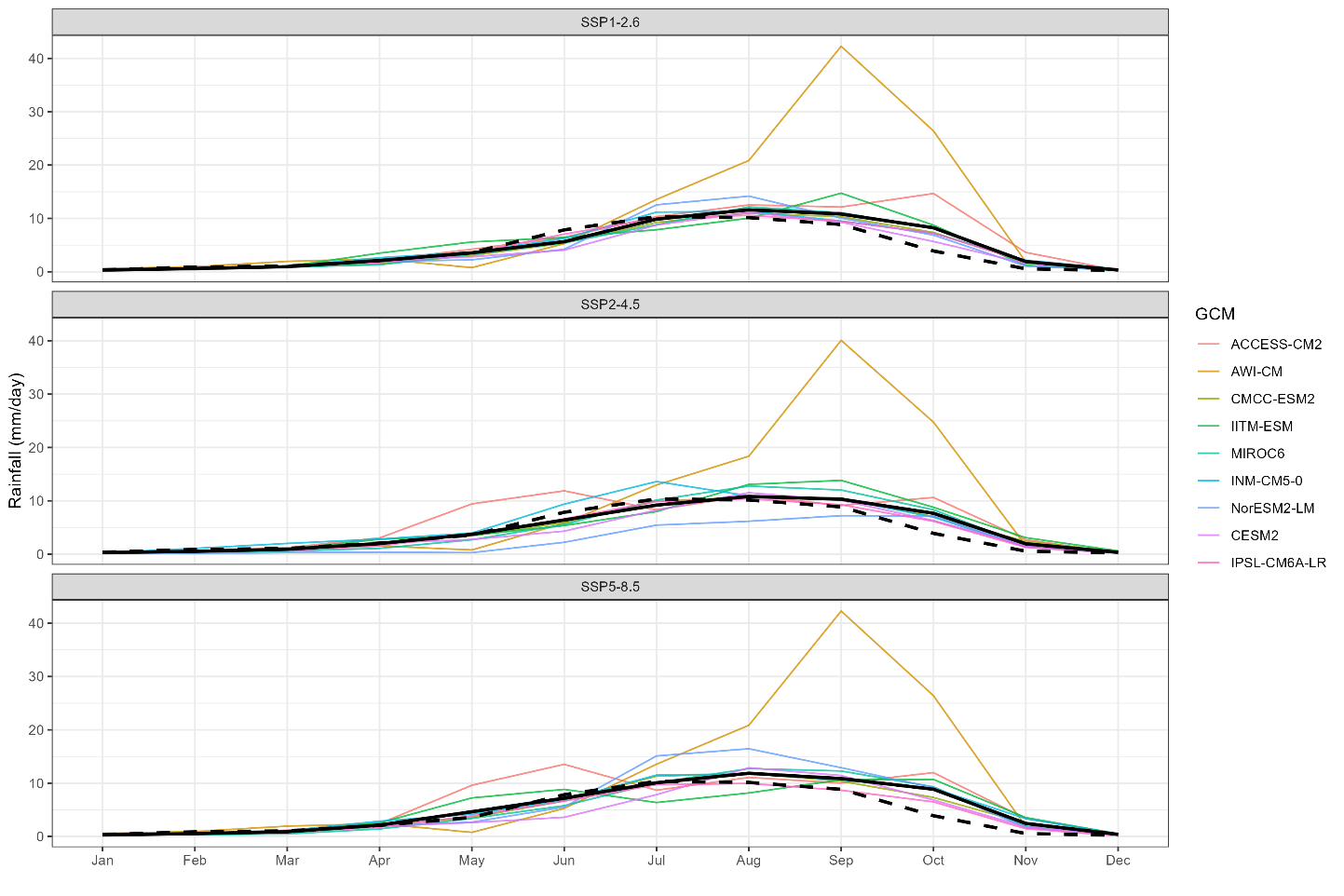


Figure S2.4- Bias-corrected rainfall projections in Kolkata. Coloured lines give the bias corrected average rainfall for each GCM under forcing scenarios SSP1-2.6, SSP2-4.5, and SSP5-8.5 during the future period 2070-2099. Solid black line shows the mean average of all GCM outputs (AWI-CM excluded) for each scenario. Dashed black represents the average temperature according to the observed reference dataset 1984-2014. Monthly average values are shown to emphasize general model differences.

**References**

1. Kobayashi S, Ota Y, Harada Y, Ebita A, Moriya M, Onoda H, et al. The JRA-55 reanalysis: General specifications and basic characteristics. Journal of the Meteorological Society of Japan. 2015;93:5–48.

2. Bi D, Dix M, Marsland S, O’farrell S, Sullivan A, Bodman R, et al. Configuration and spin-up of ACCESS-CM2, the new generation Australian Community Climate and Earth System Simulator Coupled Model. Journal of Southern Hemisphere Earth Systems Science. 2020;70:225–51.

3. Semmler T, Danilov S, Gierz P, Goessling HF, Hegewald J, Hinrichs C, et al. Simulations for CMIP6 With the AWI Climate Model AWI-CM-1-1. J Adv Model Earth Syst. 2020;12.

4. Lovato T, Peano D, Butenschön M, Materia S, Iovino D, Scoccimarro E, et al. CMIP6 Simulations With the CMCC Earth System Model (CMCC-ESM2). J Adv Model Earth Syst. 2022;14.

5. Krishnan R, Swapna P, Choudhury AD, Narayansetti S, Prajeesh AG, Singh M, et al. The IITM Earth System Model (IITM ESM) Chapter 3. 2021.

6. Tatebe H, Ogura T, Nitta T, Komuro Y, Ogochi K, Takemura T, et al. Description and basic evaluation of simulated mean state, internal variability, and climate sensitivity in MIROC6. Geosci Model Dev. 2019;12:2727–65.

7. Volodin EM, Mortikov E V., Kostrykin S V., Galin VY, Lykossov VN, Gritsun AS, et al. Simulation of the present-day climate with the climate model INMCM5. Clim Dyn. 2017;49:3715–34.

8. Seland Ø, Bentsen M, Olivié D, Toniazzo T, Gjermundsen A, Graff LS, et al. Overview of the Norwegian Earth System Model (NorESM2) and key climate response of CMIP6 DECK, historical, and scenario simulations. Geosci Model Dev. 2020;13:6165–200.

9. Danabasoglu G, Lamarque JF, Bacmeister J, Bailey DA, DuVivier AK, Edwards J, et al. The Community Earth System Model Version 2 (CESM2). J Adv Model Earth Syst. 2020;12.

10. Boucher O, Servonnat J, Albright AL, Aumont O, Balkanski Y, Bastrikov V, et al. Presentation and Evaluation of the IPSL-CM6A-LR Climate Model. J Adv Model Earth Syst. 2020;12.

11. Anandhi A, Frei A, Pierson DC, Schneiderman EM, Zion MS, Lounsbury D, et al. Examination of change factor methodologies for climate change impact assessment. Water Resour Res. 2011;47.

12. Hempel S, Frieler K, Warszawski L, Schewe J, Piontek F. A trend-preserving bias correction - The ISI-MIP approach. Earth System Dynamics. 2013;4:219–36.

13. Tabor K, Williams JW. Globally downscaled climate projections for assessing the conservation impacts of climate change. 2010.

14. Met Office, Department for Environment F and RA, Department for Business E and IS, Environment Agency. UKCP18 Guidance: Bias correction. 2018.
